# Diurnal biomarkers reveal key photosynthetic genes associated with increased oil palm yield

**DOI:** 10.1371/journal.pone.0213591

**Published:** 2019-03-11

**Authors:** Bee Keat Neoh, Yick Ching Wong, Huey Fang Teh, Theresa Lee Mei Ng, Soon Huat Tiong, Tony Eng Keong Ooi, Mohd. Zairey Md. Zain, Mohd. Amiron Ersad, Chee Keng Teh, Heng Leng Lee, Siti Khadijah Mohd Rais, See Siang Cheah, Fook Tim Chew, Harikrishna Kulaveerasingam, David Ross Appleton

**Affiliations:** 1 Sime Darby Technology Centre Sdn Bhd, Serdang, Selangor Darul Ehsan, Malaysia; 2 Sime Darby Research, Banting, Selangor Darul Ehsan, Malaysia; 3 Sime Darby Research, Pulau Carey, Selangor Darul Ehsan, Malaysia; 4 Department of Biological Sciences, Faculty of Science, National University of Singapore, Singapore; ICAR-Indian Institute of Agricultural Biotechnology, INDIA

## Abstract

To investigate limiters of photosynthate assimilation in the carbon-source limited crop, oil palm (*Elaeis guineensis* Jacq.), we measured differential metabolite, gene expression and the gas exchange in leaves in an open field for palms with distinct mesocarp oil content. We observed higher concentrations of glucose 1-phosphate, glucose 6-phosphate, sucrose 6-phosphate, and sucrose in high-oil content palms with the greatest difference being at 11:00 (*p*-value ≤0.05) immediately after the period of low morning light intensity. Three important photosynthetic genes were identified using differentially expressed gene analysis (DEGs) and were found to be significantly enriched through Gene Ontology (GO) and pathway enrichment: *chlorophyll a-b binding protein* (*CAB-13*), *photosystem I* (*PSI*), and *Ferredoxin-NADP reductase* (*FNR*), particularly for sampling points at non-peak light (11:00 and 19:00), ranging from 3.3-fold (*PSI*) and 5.6-fold (*FNR*) to 10.3-fold (*CAB-13*). Subsequent gas exchange measurements further supported increased carbon assimilation through higher level of internal CO_2_ concentration (Ci), stomatal conductance (g_s_) and transpiration rate (E) in high-oil content palms. The selection for higher expression of key photosynthesis genes together with CO_2_ assimilation under low light is likely to be important for crop improvement, in particular at full maturity and under high density planting regimes where light competition exists between palms.

## Introduction

Oil palm (*Elaeis guineensis*) is the world’s most productive oil crop on a per hectare basis and is, therefore, an important source of dietary oils and fats. Although total fruit yield, fruit bunch weight and especially mesocarp oil content are reported to be heritable [1–3], negative correlations do exist for these traits, suggesting a degree of source limitation. Also, there is a significant gap between potential and actual yield in the field caused by factors such as planting density (light competition), pollination efficiency (poor fruit formation), water and nutrient supply, field management practices, atmospheric temperature and carbon dioxide (CO_2_) concentration and photosynthetically active radiation (PAR) [4]. Previous studies on oil palm have indicated that bunch yield is positively correlated with intercepted radiation per palm. Oil palm is a C3 crop where photosynthesis is directly linked with intercepted radiation to production of sugars through photosynthesis and the Calvin cycle [5], while the photosynthetic rate of individual leaves of all crops with C3 photosynthetic pathways shows a curvilinear relationship with light intensity [6, 7]. According to Corley et al., oil palm is reported to be a source-limited crop at fruiting stage, leading to partitioning between various sinks (fruit, leaf, trunk, and root) of photosynthate to support both vegetative and reproductive growth, with the costs of maintaining the palm being the first call on resources produced by photosynthesis. The photosynthetic rate of young palms increases gradually until two months before the first bunch is harvested. The mesocarp commonly experiences lipid accumulation starting at 14–16 weeks after pollination (WAP)-and takes 22 weeks to ripen fully. The lipid production stage of mesocarp development requires significant demand on carbon sources, such as sugars produced by photosynthesis in the leaves [6, 8]. Understanding limiters of photosynthesis and carbon assimilation in oil palm and any genetic variation that may exist between different populations is therefore important for future crop improvement.

Investigating photosynthesis and its impact on yield in a large field-planted perennial crop is challenging. Meeting this challenge requires identification of the link between dynamic/short-term cellular processes to long-term yield. In addition, experimental design is also an issue as sample collection can only be performed in the open field, with no control over environmental conditions. As photosynthesis changes dynamically along with light intensity during the day, the problems of conducting accurate photosynthetic activity measurements on a large group of mature plants in a narrow time-window are not trivial. Adult palm leaf sampling and preservation for subsequent biochemical analysis was thought to provide a means of investigating the photosynthetic machinery in leaves and carbon-source accumulation in an efficient and practical manner.

A common biochemical method to observe plant photosynthesis and response to light is through investigating diurnal changes in a controlled environment. Biochemical responses of model plants throughout the diurnal cycle have been widely studied in the context of understanding plant mechanisms and reactions to certain stresses (abiotic/biotic) and also the potential for genetic modification of these processes [9–11]. Photosynthesis is typically discussed in terms of changes in light, CO_2_ assimilation and water uptake that can be related to the diurnal trend of measurable outputs such as gene expression and protein and metabolite concentrations [12–14]. According to Bläsing et al., sugar level response to light intensity in the *Arabidopsis thaliana starchless phosphoglucomutase (pgm) mutant* led to exaggerated changes of > 4,000 transcripts compared to wild-type Col-0 [14]. Most genes with exaggerated expression were observed shortly after the light was turned on and after 8 hours of continuous lighting. Furthermore, Bläsing et al. also indicated that genes required for photosynthesis were at peak at the start of the light period for sucrose synthesis that follows. In addition, the changes in sugar and transcript levels in response to light indicated a variation in the response ratio related to different light intensities.

During the daytime when the plant is actively photosynthesizing, carbon fixation in leaves supports the synthesis and export of sucrose to the remainder of the plant for storage and to support metabolism and growth. At night, the plant becomes a net consumer of carbon [15]. As sugars are the precursors for several metabolic processes such as glycolysis and the pentose phosphate pathway, the efficiency of plant metabolism is likely to be affected by the concentration of sugars generated from photosynthesis. The supply of sucrose coming from tomato leaves to fruits has been related to compositional changes in leaves and metabolic processes in fruits [16]. Similarly, in Arabidopsis higher light intensity gave a two-fold increase in seed yield, 40% increase in mass per seed and a 60% increase in oil per seed [17]. In a recent study on adult oil palm photosynthesis, the optimum irradiance, maximum photosynthetic oxygen evolution rate and photosynthetic efficiency are maximum at midday [18].

In this study, we investigated metabolite concentrations, gene expression changes and leaf gas exchange in two populations of mature oil palms with differing -mesocarp oil content throughout the diurnal cycle (sunrise, mid-morning, mid-afternoon, and sunset) to investigate how dynamic changes in photosynthesis may be related to oil yield.

## Materials and methods

### Plant materials

Twenty *tenera* palms derived from *Banting Dura* X AVROS *pisifera* crosses (planted October 2005) were sampled from Sime Darby Research Sdn Bhd breeding trial plot in East Estate, Carey Island, Malaysia (2°51’08”N; 101°23’03”E). Sime Darby Research Sdn Bhd and East Estate of Sime Darby Plantation Bhd. have granted the permission for this research and does not involved any endangered species. Palms were selected based on mesocarp oil content determined by the Bunch Analysis Laboratory, Banting, Malaysia, according to standard practice with slight modification [1, 19, 20]. Results from three analyses per palm sampled over a three-year period (2010 to 2012) were averaged to determine fruit bunch composition. The mean mesocarp oil content (w/w, oil to dry mesocarp), was 79.3 ± 0.9% for the high-mesocarp oil content group (HY; n = 10) and 74.6 ± 2.2% for the low-mesocarp oil content group (LY; n = 10), both groups being a subset of a population with same age pedigree and planted in the same location with overall mean 77.3 ± 1.9% (n = 60). Besides mesocarp oil content, other components such as bunch and mesocarp weight, total oil content in bunch and palm were also significantly higher in HY palms (S1 Table). Vegetative measurements were also conducted at palm age of five years old, including frond length and weight; rachis weight, thickness and width; number of pinnae and trunk height. No significant difference in any of the vegetative measurements was noted between the HY and LY groups (S1 Table), and thus the physical characteristics of both palm groups were considered unbiased.

### Sampling

Counting from the newest frond with partially open leaves (frond number 1), frond number 12 was selected for sampling. In oil palm, fronds are arranged in eight spiral, and frond 12 is situated on the second crown (layer), similar to frond number 17, another commonly studied frond in oil palm investigations. As oil palm leaves appear at regular intervals in the double spiral [21], the shading of frond 12 in the second crown from fronds 2 and 4 (fully erect) in the first crown is minimal [22]. With at planting density of 136 palms per hectare, the overlapping of frond 12 with other palms was insignificant. Frond 12 was selected for this study because it has fully expanded leaves, receives maximum radiation [23], similar photosynthesis rate (no significant difference among fronds 4 to 30) [24] and can be considered physiologically stable. Previous unpublished data (S2 Table) shows the metabolite concentrations of fronds 12 and 17 were similar over a period of months. In this study, frond 12 of each palm was sampled at five-time points throughout the diurnal cycle (07:00, 11:00, 15:00, 19:00 and finally, 07:00 on the following day). Three random leaflets were sampled at each time point from the same frond. Generally, a fully extended frond comprises 250–300 leaflets; therefore, the sampling of 15 leaflets over the 24-h period was considered not enough to affect the photosynthesis mechanism of the palm or to cause metabolic changes in the frond overall. As the palms were in an open field in an oil palm estate, no sampling was conducted at night due to safety reasons. To minimize variation in results due to sampling it was decided that 1) leaves were to be sampled within a 30-min window for each time point, 2) time points were set to accord with sunrise, mid-morning (before peak light), mid-afternoon and just before sunset. Solar radiation measurements (Wm-^2^) were obtained from the local weather station at nearby Dusun Durian Estate (15 km from the sampling plot).

Leaf samples were snap frozen in liquid nitrogen immediately after sampling, then transported back to the laboratory and kept in a -80°C freezer before maceration. For each palm and time point, the three leaflets were combined and ground with liquid nitrogen using mortar and pestle. Powdered samples were then kept in a -80°C freezer until further use.

### Metabolite extraction

Powdered leaf tissues (500 mg) were extracted using 5 mL of 80% (v/v) methanol in water with 0.1% BHT. Ribitol (2 mg mL^-1^) was added to the tissue as an internal standard. The mixture was shaken for 30 s using a vortex mixer and incubated at 4°C for 5 min at 500 rpm, followed by sonication for 5 min at 10°C. The incubation and sonication procedures were repeated three times, followed by centrifuging at 4°C and 4000 rpm for 20 min. The methanol extract was transferred into four pre-weighed new tubes (1 mL each). The extracts were dried using a centrifugal evaporator (Genevac EZ2- Elite, United Kingdom) and the weights recorded. All extracts were kept dry at -80°C until further use. The metabolites, including carotene, chlorophyll, amino acids, organic acids, sugars, sugar phosphates, nucleotides, nucleosides and polyamines, were analysed using gas chromatography-mass spectrometry [25], liquid chromatography-mass spectrometry (LC-MS) and capillary electrophoresis-mass spectrometry [26], following the methods reported in Neoh et al., 2013.

### Photosynthetic targeted LC-MS analysis

Chromatography was conducted using an Acquity UPLC HSS T3 column (1.8um, 2.1 x 100 mm; Waters, Massachusetts, United States). The column and samples were maintained at 30°C and 4°C, respectively. The liquid chromatography mobile phases used were 0.1% formic acid in water (solvent A) and 0.1% formic acid in ethanol (solvent B). The flow rate was 0.6 mL min^-1^. The elution gradient was as follows: initial to 2 min with isocratic elution of 95% solvent B; 2–8 min linear gradient to 100% solvent B and hold for 2 min; 10–10.1 min linear gradient to 95% solvent B and hold to 5 min. The injection volume was 3 μL. The mass spectrometer was operated in positive mode using the electrospray ionization technique with multiple reactions monitoring. The capillary voltage was set to 3.5 kV, desolvation gas set at 1000 L h^-1^ at 450°C. The collision gas flow was set at 0.15 mL min^-1^.

### Total starch content

The total starch content was measured using a Tecan Plate Reader Infinite M200 (Männedorf, Switzerland) at 528 nm by using hot ethanol extraction and enzymatic assay [27].

### RNA extraction

Powdered leaf tissue (0.4–0.5 g) was extracted using the RNA extraction kit, NucleoSpin RNA Plant (Macherey-Nagel, Düren, Germany). After extraction, the samples were analyzed using Bioanalyzer 2100 (Agilent, California, United States). The samples with RIN number 6 or higher were sent for sequencing. A total of 2–3 μg of total RNA was sent for sequencing.

### Gene expression study

Gene expression was profiled using the Illumina Hiseq2000 platform, (San Diego, United States) outsourced to BGI-TECH, Hong Kong. A transcript library was constructed for all leaf samples, and all transcripts were sequenced using 100 bp pair-end sequencing technology. The sequenced transcripts were annotated using the oil palm reference transcripts (European Nucleotide Archive (ENA), accession number(s): LM611910-LM643713).

### Gene ontology and pathway enrichment analysis

The differential analysis compared the differential transcripts between HY and LY populations and at different collection time points. Differentially expressed genes (DEGs) were identified through NOISeq with the threshold q-value (Q) ≥ 0.8. In addition, a cluster analysis of gene expression patterns of DEGs was performed using cluster and Java Treeview software. The identified DEGs were then subjected to Gene Ontology (GO) function analysis. After GO function analysis, GO enrichment analysis yielded all GO terms in the list of DEGs that were significantly enriched, to compare the GO terms to a genome background and also to filter the DEGs that correspond to specific biological functions. We first mapped all the DEGs with GO terms in the *http*:*//www*.*geneontology*.*org/* database, calculating the gene numbers for every term and then identifying the significantly enriched GO terms in the input list of DEGs using a hypergeometric test. The *p*-value for GO analysis was calculated using Bonferroni Correction, and the significantly enriched GO terms in the DEGs threshold was set with a corrected *p*-value ≤ 0.05. The DEGs were also subjected to KEGG pathway enrichment analysis. The threshold of the enrichment was set with a *p*-value ≤ 0.05.

### Quantitative real-time PCR

Validation of the gene expression was performed using quantitative real-time PCR (qPCR) (S1 Fig). Two micrograms of total RNA from the same leaf samples used in sequencing were used in a reverse transcription reaction using the Tectro cDNA Synthesis Kit with standard conditions as recommended by the manufacturer (BIOLINE, London, United Kingdom). The first strand cDNA was synthesized using random hexamer primers. Specific primers were designed using the Primer Premier 5.0 software. The qPCR was performed using a 7500 Real-Time PCR System (Applied Biosystems, California, and United States). The reaction mixture and cycling conditions were based on optimized conditions of the SensiFAST SYBR Hi-ROX Kit suggested by BIOLINE, the UK with 95°C (2 min) for one cycle and followed by 95°C (5 s) and 55–60°C (30 s), for 40 cycles. Relative expression of each transcript was normalized against three reference genes (*Cyp2*, *Slu7*, and *GRAS*) and analyzed using the qBase Plus 2.0 software [28].

### Gas exchange

Leaf gas exchange measurements were carried out at 7:00, 11:00 and 15:00, where representative samples of 5 HY and 5 LY palms were taken within one hour of each time point. A single measurement was collected from three middle leaflets of mid cut-frond using the infrared gas analyzer LICOR 6400 Portable Photosynthesis System (IRGA: LICOR Inc., Lincoln, NE, USA) by placing the lamina of fully expanded leaflets in a leaf cuvette supported by a tripod stand. Standard optimal cuvette conditions with modification [29] were used and set to 400 μmol mol^-1^ CO_2_, 25°C leaf temperature and 60% relative humidity while photosynthetic photon flux density to 100 (at 7:00), 1,000 (at 11:00) and 1200 μmol m^-2^s^-1^ (at 15:00) based on environmental light intensity.

### Multivariate and statistical analyses

Principle component analysis (PCA) and orthogonal partial least squares (OPLS) using Simca-P version 1.3 (Umetrics) were used to identify metabolite cluster according to the palm categories (HY and LY) and sampling time points. The t-test algorithm of Excel 2000 (Microsoft) was used to determine significance. Two ranges of *p*-values were reported: *p*-value ≤ 0.05, and *p*-value ≤ 0.10 for cases where the limited feasible sample size and inherent variability in field sample measurements reduced the experimental power possible.

## Results

**Fig 1:**
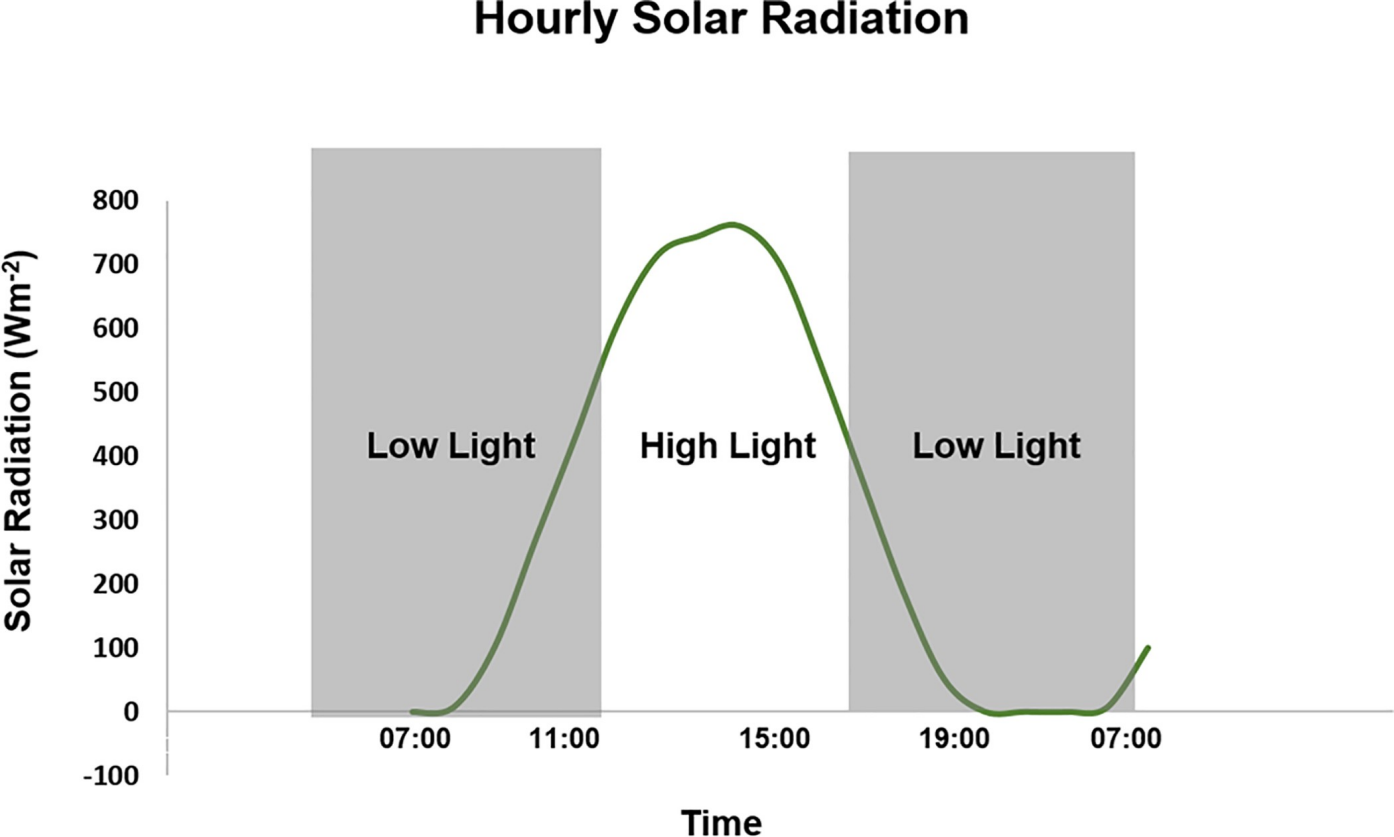
Solar radiation (Wm^-2^) reading based on sampling time, collected from Dusun Durian, Selangor.

Diurnal light intensities were recorded according to solar radiation at the Dusun Durian Estate weather station for May 2016 (Fig 1). The record showed the light intensity increased above zero at around 07:00 with 7 Wm^-2^, rising to a maximum of 760 Wm^-2^ at 14:00. Light intensity then decreased to 0.5 Wm^-2^ by 20:00. In this study, oil palm leaf samples were collected at five different time points associated with likely boundary points in photosynthetic activity due to light intensity changes throughout the day: 07:00—commencement of photosynthesis; 11:00—near the boundary at the end of the low light morning period; 15:00—at the end of peak light at midday; and 19:00—end of day light intensity minimum before dark. A fifth sampling point was taken at 07:00 the following day to complete a full 24 h diurnal cycle (Fig 1).

### Differential metabolite concentrations across different light intensities

Leaf metabolite analysis in this study was focused on 156 individual metabolites representing primary metabolites, including sugars, sugar phosphates, amino acids, organic acids, nucleotides, nucleosides, mono- and polyamines, hormones, carotenes, and chlorophylls. The selection of metabolites was based on the metabolites that have been reported to show rapid changes during the diurnal cycle of the starchless *pgm* mutant *Arabidopsis* [15]. All metabolites were compared between HY and LY throughout the diurnal cycle using PCA (S2 Fig), OPLS (S3 Fig), and t-test (S3 Table). At all sampling time points, serine (Ser) was found to be more concentrated in the HY group, as supported by *p*-values ≤ 0.05 for 07:00, 15:00 and 19:00. In plants, serine is involved in both photorespiratory glycolate and non-photorespiratory phosphorylated pathways, and is required for cell proliferation, and biosynthesis of amino acids, nitrogenous bases, phospholipids, and sphingolipids[30]. According to Ros et al., the existence of different pathways of serine biosynthesis complicates the understanding of serine homeostasis, and due to the lack of functional investigation in current literature, serine correlation to HY palms is not further discussed here. Another metabolite identified in PCA and OPLS analyses as being more abundant in the LY group was tyramine, however only significant at Day 2 07:00 with *p*-value ≤ 0.05.

**Fig 2:**
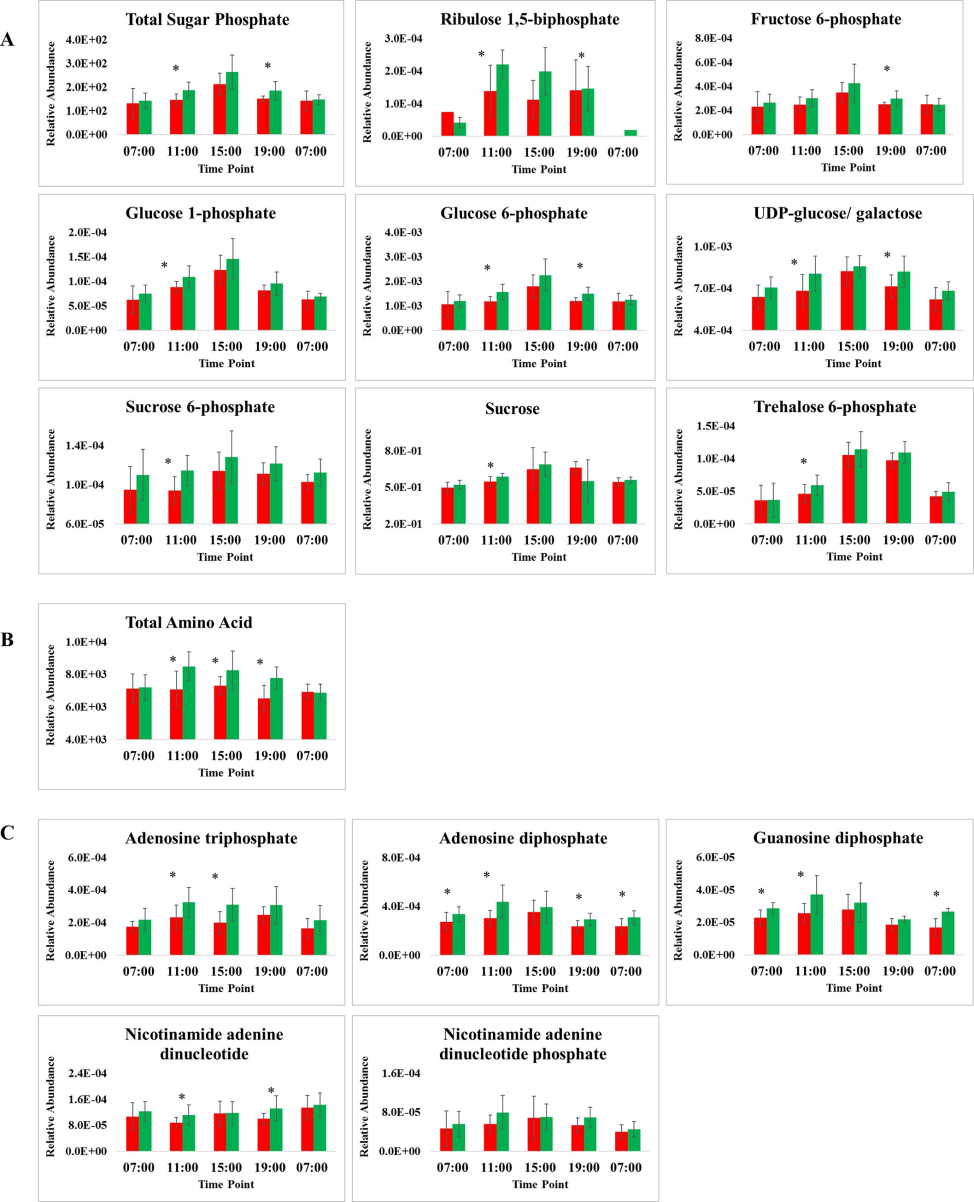
**Diurnal trend and comparison of A) sucrose and sugar phosphates, B) total amino acids, and C) nucleotides that showed higher concentration in HY palm throughout the circadian cycle.** Green bar represents the average of HY (n = 10); Red bar represents the average of LY (n = 10) collected in the open field. The concentration of metabolites was quantified by relative abundance to the internal standard, Ribitol (2 mg mL^-1^). * represents a significant difference between HY and LY with *p*-value ≤ 0.10. The concentration of metabolites was quantified by relative abundance compared to the internal standard, Ribitol (2 mg mL^-1^).

Analysis of total leaf starch content indicated no significant difference between HY and LY at all time points (S4 Table). Metabolites such as sugars and sugar phosphates displayed a diurnal trend that was low during low light intensity (07:00–11:00), highest at 15:00, and decreased as light intensity waned towards 19:00 (Fig 2A). Using OPLS analysis, sugars and sugars phosphates were found to have high predictive loading values (p(corr)), although less variation in mean between HY and LY. Using t-test, differences in total sugar phosphates between HY and LY had a *p*-value ≤ 0.05 (Total abundance: HY = 187.7 ± 32.5; LY = 145.7 ± 26.3) at 11:00 and 19:00 (HY = 184.9 ± 38.3; LY = 150.7 ± 11.8), while at 15:00, the total abundance of HY was 263.8 ± 71.3 and LY 211.3 ± 47.2 with a non-significant *p*-value of 0.11. The diurnal trend of individual sugar phosphates analysed [sucrose 6-phosphate (S6P), trehalose 6-phosphate (Tre6P), glucose 1-phosphate (G1P), glucose 6-phosphate (G6P), fructose 6-phosphate (F6P), uridine diphosphaphate -glucose/ galactose (UDP-glu/gal)] were similar to total sugar phosphates with higher concentration in HY palms, all being significantly different at 11:00 (*p*-value ≤ 0.10) with the exception of F6P. HY palms were also observed to have higher concentration of sucrose compared to LY at 11:00 (Fig 2A).

Accumulation of leaf amino acids was observed early in the day, but decreased after 11:00. Total amino acids (Fig 2B) were observed to be consistently high in HY palms with a *p*-value ≤ 0.05 at 11:00, 0.08 at 15:00 and 0.009 at 19:00. In addition to serine, several amino acids, such as citrulline, threonine, 2-methylserine, arginine and asparagine (S3 Table) were also more highly accumulated in HY throughout the diurnal cycle compared to the LY group.

Fig 2C shows a general trend of higher nucleotides and their derivatives, such as adenosine triphosphate (ATP), in the HY samples and again were significantly different at 11:00 (HY = 3.2 x 10^−4^ ± 0.9 x 10^−4^; LY = 2.3 x 10^−4^ ± 0.8 x 10^−4^) and at 15:00 (HY = 3.1 x 10^−4^ ± 1.0 x 10^−4^; LY = 2.0 x 10^−4^ ± 0.7 x 10^−4^) with a *p*-value ≤ 0.10. In addition to ATP, adenosine diphosphate (ADP), guanosine diphosphate (GDP) and nicotinamide adenine dinucleotide were found to be at higher concentrations in HY palm, especially during low light. The diurnal trends of ADP and GDP were observed to be similar, with concentrations peaking in HY at 11:00 and reaching minimum at 19:00, whereas optimum concentration peaked at 15:00 for LY and decreased after that. For ADP a significant difference (*p*-value ≤ 0.10) between HY and LY was found at 07:00 (HY = 3.4 x 10^−4^ ± 0.6 x 10^−4^; LY = 2.7.0 x 10^−4^ ± 0.8 x 10^−4^), 11:00 (HY = 4.4 x 10^−4^ ± 1.4 x 10^−4^; LY = 3.0 x 10^−4^ ± 0.6 x 10^−4^), 19:00 (HY = 2.9x 10^−4^ ± 0.5 x 10^−4^, LY = 2.3 x 10^−4^ ± 0.5 x 10^−4^,) and at 07:00 the following day (HY = 3.1 x 10^−4^ ± 0.6 x 10^−4^; LY = 2.4 x 10^−4^ ± 0.6 x 10^−4^,). For GDP, significant differences (*p*-value ≤ 0.05) between HY and LY were found at 07:00 both days (07:00-Day 1: HY = 2.9 x 10^−5^ ± 0.4 x 10^−5^; LY = 2.3 x 10^−5^ ± 0.5 x 10^−5^, 07:00-Day 2: HY = 2.7 x 10^−5^ ± 0.2 x 10^−5^; LY = 1.7 x 10^−5^ ± 0.5 x 10^−5^) and at 11:00 (HY = 3.7 x 10^−5^ ± 1.2 x 10^−5^; LY = 2.6 x 10^−5^ ± 0.6 x 10^−5^)

No significant difference in chlorophyll content was observed between HY and LY throughout the diurnal cycle.

### Differentially expressed gene ontology and enrichment analysis

**Fig 3:**
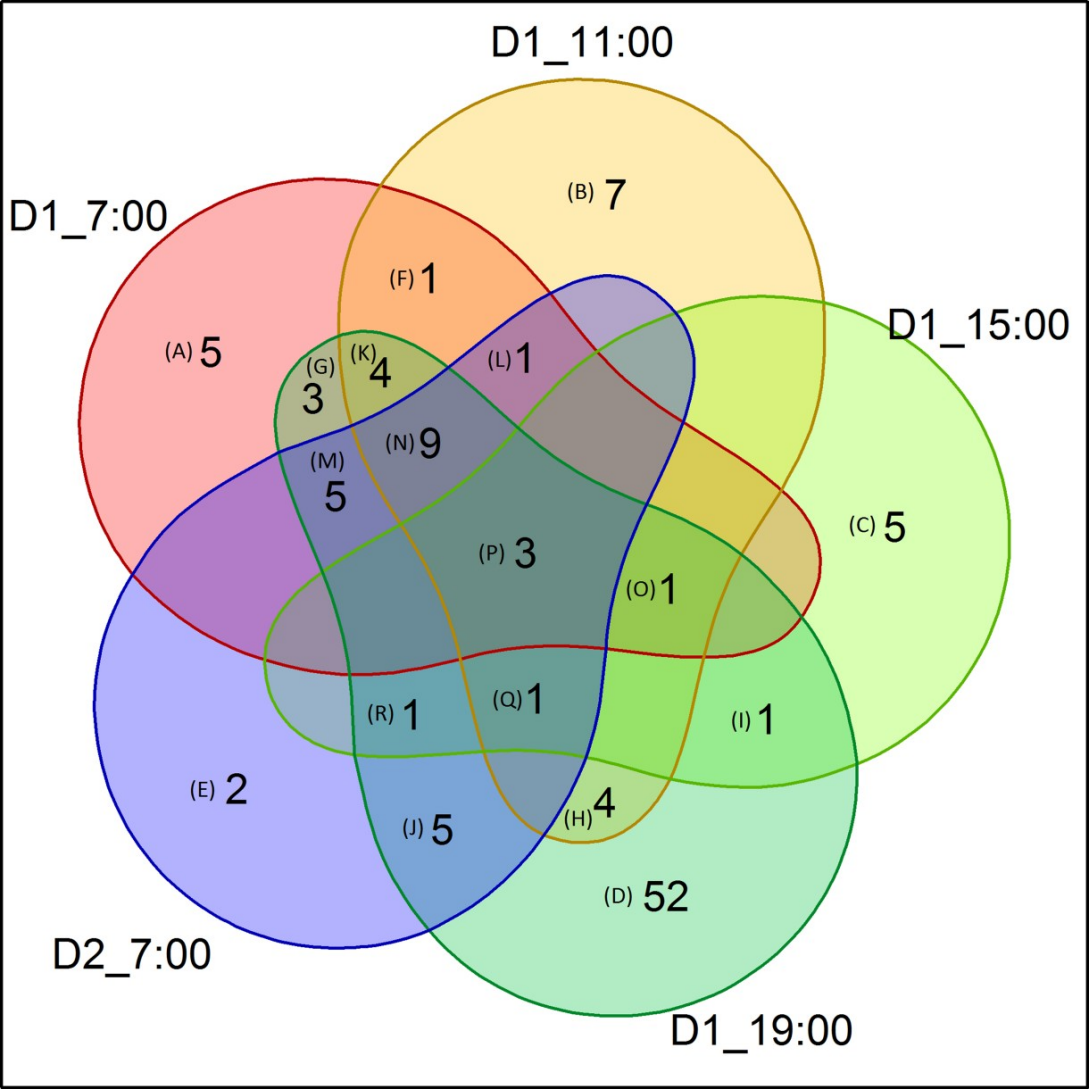
Differentially expressed genes (DEGs) comparison analysis of all 5 time points. Q value ≥ 0.8. D1_07:00, D2_11:00, D1_15:00 and D1_19:00 are the sampling time points within the same day. D2_07:00 is the sampling time point the next day. The numbers in the diagram indicate the total number of transcripts in that cluster. The annotation of the transcript/s within a cluster are listed in S6 Table.

A total of 191 DEGs were identified from transcriptome analysis comparing HY and LY oil palm leaf samples across the five diurnal time points (S5 Table). A total of 32 DEGs were identified at time point 07:00, 31 DEGs at 11:00, 12 DEG at 15:00, 89 DEGs at 19:00 and lastly 27 DEGs at 07:00 the next day. From the DEGs analysis, most of the DEGs between HY and LY were identified at low light time points 07:00, 11:00, 19:00 and 07:00 the next day (93.7%) compared to high light, 15:00 (6.3%). The Venn diagram in Fig 3 shows that only three transcripts were differentially expressed across all time points (*Homeobox-leucine zipper* and two unannotated transcripts). *Homeobox-leucine zipper* found to be down-regulated in HY oil palms across all time points and other two unannotated (isotig56650 and isotig69058) were up-regulated across all time points (S6 Table). A total of 11 transcripts were differentially expressed across four time points in cluster N, O and Q (Fig 3), including transcripts coded for photosynthesis related gene *Ferredoxin-NADP reductase* (*FNR*), *Tyrosine-sulfated glycopeptide receptor 1* and *Iron sulfur cluster assembly protein 1*. Out of 11 DEGs in these clusters, only *FNR* was significantly expressed in HY palms across low light and high light (7:00, 11:00, 15:00 and 19:00). Cluster K, L, M and R contained total of 11 DEGs. The transcripts in these clusters included C*hlorophyll a-b binding protein subunit-13* (*CAB-13)* and 2-oxoglutarate/malate carrier protein. *CAB-13* was found to be differentially expressed at 07:00, 11:00 and 19:00. Of all the time points, 19:00 had the highest number of unique DEGs (52 in total). Out of total 191 DEGs, only 110 were unique genes, of which 57—were up-regulated and 53 down-regulated in HY oil palm samples. Sixty-three genes were annotated using the UniProt databases (S5 Table).

In order to functionally classify the 191 DEGs, they were mapped with GO terms through the gene ontology database (http://www.geneontology.org/). Successfully mapped genes were then subjected to GO enrichment (S7 Table). A total of 23 GO terms were enriched by the analysis: biological processes (8), cellular components (14) and molecular functions (1). Five of the enriched GOs were classified under various metabolic and biosynthesis processes based on biological processes (Table 1), where these GO terms consisted of most of the DEGs classified in biological processes. For cellular components, most of the differentially expressed genes were enriched in the plastid (GO0009536) across three time points (07:00, 11:00 and 19:00). Almost all enriched GOs were found at the lower light intensity time points of 07:00, 11:00 and 19:00 while only one GO was enriched at 15:00, the high light time point (GO0003824). No GO term was found significantly enriched at time point 07:00 the following day. However, from the log_2_ ratio of the expression at 07:00 the next day, some of these DEGs showed differential expression in HY oil palm (log_2_ ratio from 2.95 to 7.75) when the log_2_ ratio of expression was set at two and these included three photosynthesis related genes such as *CAB-13*, *FNR and Photosystem I reaction centre subunit XI* (*PSI)*.

**Table 1:** GO enrichment analysis of all differentially expressed genes in high- and low-yielding oil palms. Cut off threshold, *p*-value ≤ 0.05. Ticks indicate the presence of GO terms in specific time point.

				Time point				
Gene Ontology	Annotation	Up regulation	Down regulation	07:00	11:00	15:00	19:00	Ontology
GO:0009987	cellular process	6	0		√			biological process
GO:0044237	cellular metabolic process	16	4	√	√		√	biological process
GO:0044238	primary metabolic process	5	0	√	√			biological process
GO:0044249	cellular biosynthetic process	4	0		√			biological process
GO:0044699	single-organism process	4	0		√			biological process
GO:0051704	multi-organism process	4	0		√			biological process
GO:0071704	organic substance metabolic process	5	0	√	√			biological process
GO:1901576	organic substance biosynthetic process	4	0		√			biological process
GO:0005622	intracellular	9	1		√			cellular component
GO:0005623	cell	9	1		√			cellular component
GO:0005737	cytoplasm	9	1		√			cellular component
GO:0009507	chloroplast	6	0	√	√			cellular component
GO:0009536	plastid	12	1	√	√		√	cellular component
GO:0016020	membrane	4	0		√			cellular component
GO:0032991	macromolecular complex	7	0				√	cellular component
GO:0043226	organelle	8	1		√			cellular component
GO:0043227	membrane-bounded organelle	8	1		√			cellular component
GO:0043229	intracellular organelle	8	1		√			cellular component
GO:0043231	intracellular membrane-bounded organelle	8	1		√			cellular component
GO:0044424	intracellular part	9	1		√			cellular component
GO:0044444	cytoplasmic part	9	1		√			cellular component
GO:0044464	cell part	9	1		√			cellular component
GO:0003824	catalytic activity	5	0			√		molecular function

**Fig 4:**
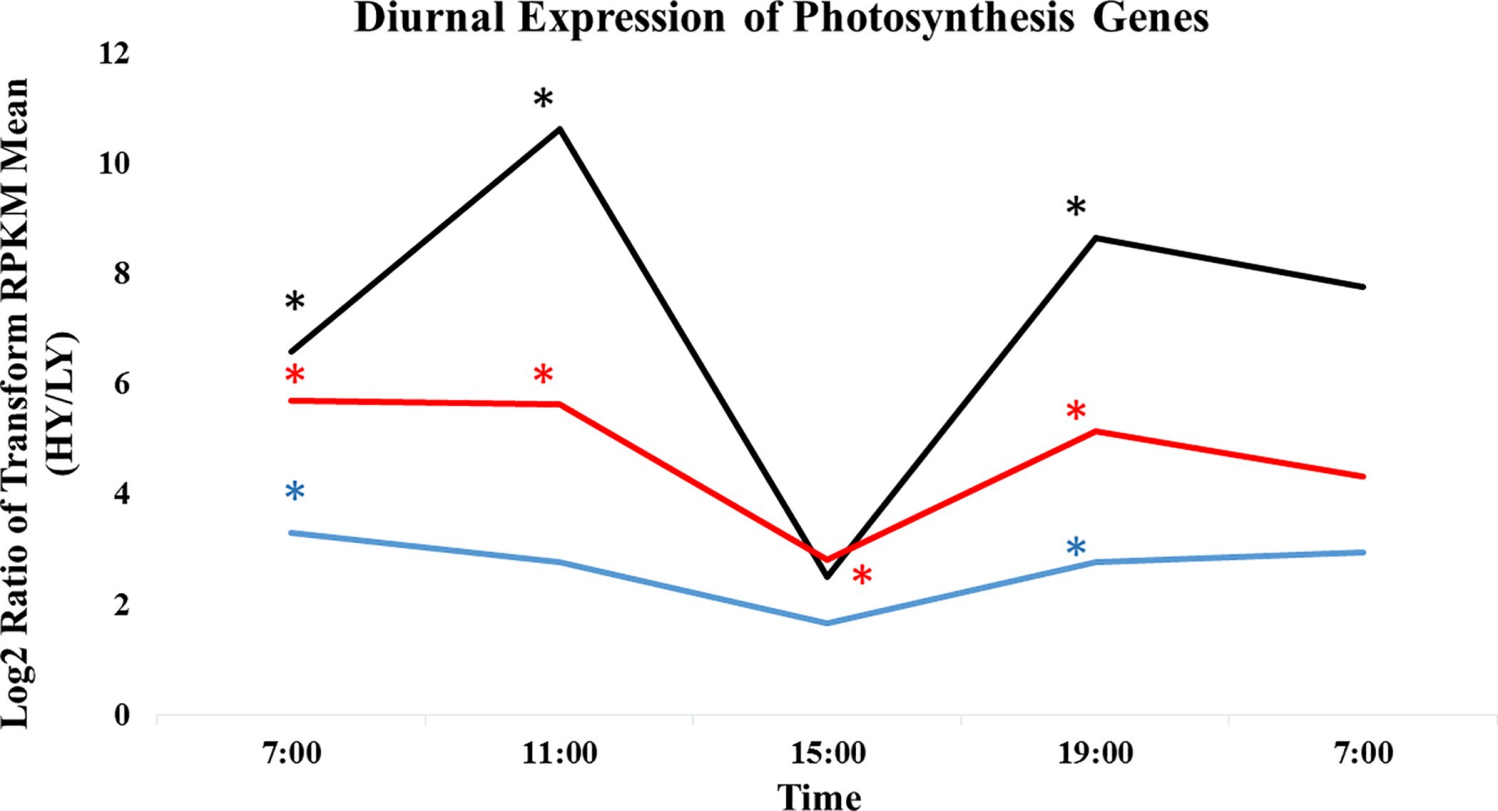
Differential expression of three photosynthesis related genes, *Photosystem I* (*PSI*), *Chlorophyll a-b binding protein 13* (*CAB-13*) and *Ferredoxin- NADP reductase* (*FNR*) in HY and LY palms (n = 20) using RNAseq. * Significant differences between HY and LY oil palm Log_2_ ratio ≥ 2, Q ≥ 0.80 is explained by the probability of equivalent expression in Noiseq [31]. Blue line represents the expression of *PSI*, black *CAB-13*, red *FNR*.

To understand the pathways that involve the 191 DEGs, we carried out KEGG pathway enrichment analysis. A total of ten pathways were enriched in the analysis (Table 2). Photosynthesis (ko00195) was the most enriched pathway in the analysis, being enriched at three time points 07:00, 11:00 and 19:00. Photosynthesis-antena protein (ko00196) and Nitrogen metabolism (ko00910) were the second most enriched pathways. Photosynthesis-antena protein was enriched at 07:00, 11:00 and 19:00. The nitrogen metabolism pathway was enriched at 07:00, 19:00 and 07:00 the next day. This was followed by plant hormone signal transduction (ko4075) at 11:00 and 19:00. All the four most enriched pathways were found at low light time points of 07:00, 11:00 and 19:00. Only photosynthesis (ko00195) was found to be enriched across low light and high light time points. Pathways such as monoterpenoid biosynthesis (ko00902), sesquiterpenoid and triterpenoid biosynthesis (ko00909), Alpha-Linolenic acid metabolism (ko00592), Cutin, suberine and wax biosynthesis (ko00073) and Terpenoid backbone biosynthesis (ko00900) were found to be enriched only at high light, 15:00. The two most enriched pathways (Photosynthesis and Photosynthesis-antena protein), three genes; *CAB-13*, *FNR* and *PSI* were found to be differentially expressed in HY oil palm across several time points particularly at sampling time points associated with low light (07:00, 11:00 and 19:00), only *FNR* was found to be differentially expressed across low and high light intensities (Fig 4). The differences in expression observed were also validated using qPCR (S1 Fig).

**Table 2:** Pathways enrichment analysis of all differentially expressed genes in high-yielding oil palms. Cut off threshold, *p*-value ≤ 0.05. Ticks indicate the enriched pathway in specific time point.

		Time point				
Pathway ID	Annotation	07:00	11:00	15:00	19:00	07:00 (next day)
ko00195	Photosynthesis	√	√	√	√	
ko00196	Photosynthesis—antenna proteins	√	√		√	
ko00910	Nitrogen metabolism	√			√	√
ko04075	Plant hormone signal transduction		√		√	
ko00902	Monoterpenoid biosynthesis			√		
ko00909	Sesquiterpenoid and triterpenoid biosynthesis			√		
ko00592	alpha-Linolenic acid metabolism			√		
ko00073	Cutin, suberine and wax biosynthesis			√		
ko00900	Terpenoid backbone biosynthesis			√		
ko00250	Alanine, aspartate and glutamate metabolism				√	

### Oil palm leaf gas exchange

**Fig 5:**
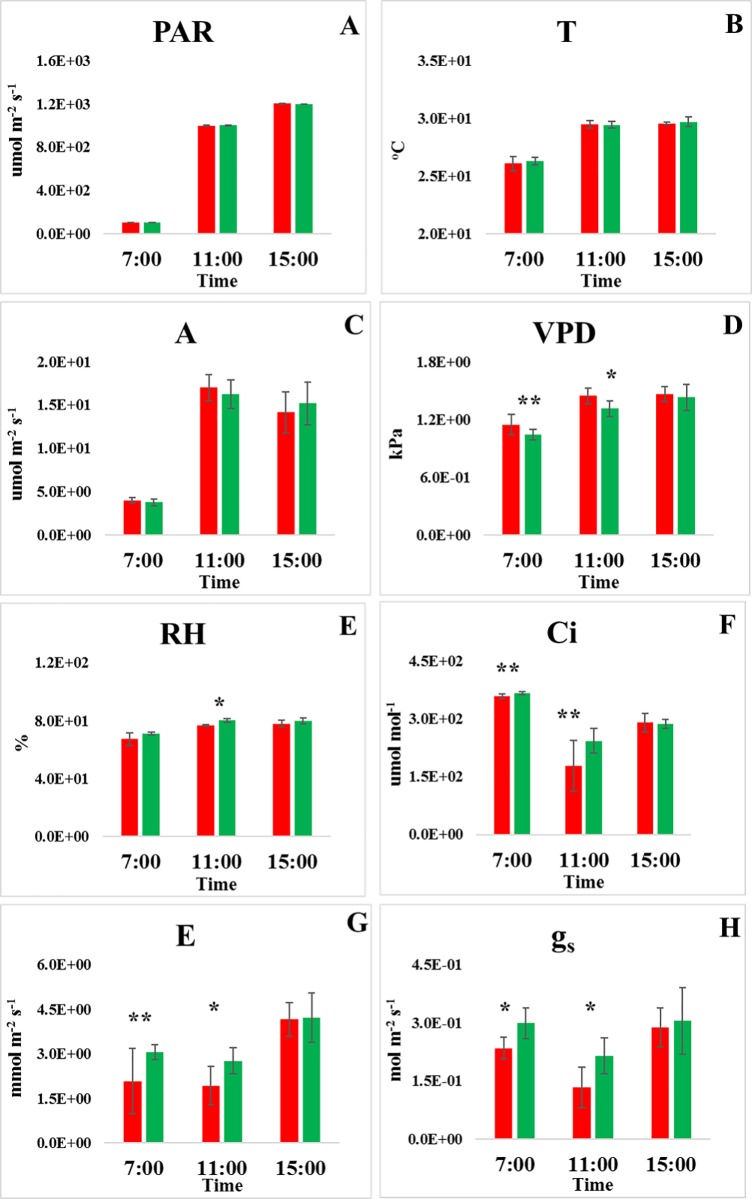
**Diurnal changes on (A) photosynthetically active radiation, PAR, (B) leaf temperature, T, (C) net photosynthesis, A, (D) leaf air vapour pressure deficit, VPD, (E) relative humidity, RH, (F) internal CO**_**2**_ **concentration, Ci, (G) transpiration rate, E and (H) stomatal conductance, g**_**s**_ **of oil palms measured in field.** Green bar represents the average of HY (n = 5); Red bar represents the average of LY (n = 5); * represents a significant difference between HY and LY with *p*-value ≤ 0.05. ** represents a significant difference between HY and LY with *p*-value ≤ 0.10.

Photosynthetically active radiation (PAR) and leaf temperature (T) showed similar diurnal trends (Fig 5) with clear increment from 7:00 to 11:00 but only slight increment observed from 11:00 to 15:00. The most significant differences between HY and LY were noted at 11:00, in particular for stomatal conductance (gs), transpiration rate (E), relative humidity (RH) and leaf air vapour pressure deficit (VPD), all with p-value ≤ 0.05. The trend of net photosynthesis (A) was almost as integrated as PAR, except there was minor decreasing after 11:00. The values of PAR, T and A were recorded for HY and LY, and there were no differences monitored. However, the relative humidity (RH) and leaf air vapour pressure deficit (VPD) that shared the similar trend as PAR and T, were found to be significant at 11:00 (*p*-value ≤ 0.05). The negative trend was observed on internal CO_2_ concentration (Ci), stomatal conductance (g_s_) and transpiration rate (E) where the lowest value was found at 11:00.

## Discussion

Improvement of photosynthetic capacity or activity to increase overall oil palm yield has long been discussed, but to pinpoint the critical rate-limiting processes, we must understand the flow of photosynthesis starting from light interception to gene expression, followed by sugar production and finally the carbon and nitrogen translocation to the mesocarp for oil biosynthesis. As oil palm is a perennial crop, it is challenging to relate short-term cellular processes to long-term oil yield trends, especially comparing leaf biochemical processes and fruit development. The long-term partitioning of carbon and other metabolites between vegetative and reproductive growth can be confounding when attempting to relate instantaneous biochemical or physiological measurements. Moreover, carbon storage in the trunk represents a comparatively large buffer, likely compensating for some of the short-term carbohydrate and starch fluctuations when photosynthesis is insufficient to support immediate plant demand. In an open field, variables such as the number, age, and size of bunches will differ among palms, along with the number of fronds, height and amount of light intercepted. By adopting what is essentially a bulking strategy based on oil yield (the key trait of interest), it is possible to screen for consistent differences between palms in terms of yield performance and fruit characteristics. These differences can be used to identify markers that could potentially be used to screen and select for higher yield.

A high throughput metabolomics and transcriptomics approach was adopted to investigate the relationship between diurnal leaf biochemistry and yield performance of oil palm in open field. Oil palm samples were grouped based on long- term observations of mesocarp oil content as this is one of the most heritable traits contributing to overall yield [1].

Sampling time points for metabolites and expression studies were fixed at every four hours and selected to represent key boundary points in photosynthetic activity, starting from 07:00 (sunrise). As expected, gene expression varied throughout the day with different clusters of genes peaking at different time points. Also as expected, metabolites that exhibited a distinct diurnal pattern were sugars, sugar phosphates and nucleotides, with low concentrations at the start of the day (07:00), increasing toward the end of peak light intensity (15:00) and then decreasing as light intensity reduced (19:00) (Fig 2). Diurnal changes were especially evident for genes assigned to trehalose metabolism, nutrient uptake and assimilation, and redox regulation as well as affecting other sectors of metabolism including glycolysis, gluconeogenesis, the tricarboxylic acid cycle, metabolisms of amino acid, lipid, nucleotide, secondary metabolites, polyamine, tetrapyrole synthesis, starch and sucrose metabolism and, most of all, photosynthesis [14].

**Fig 6:**
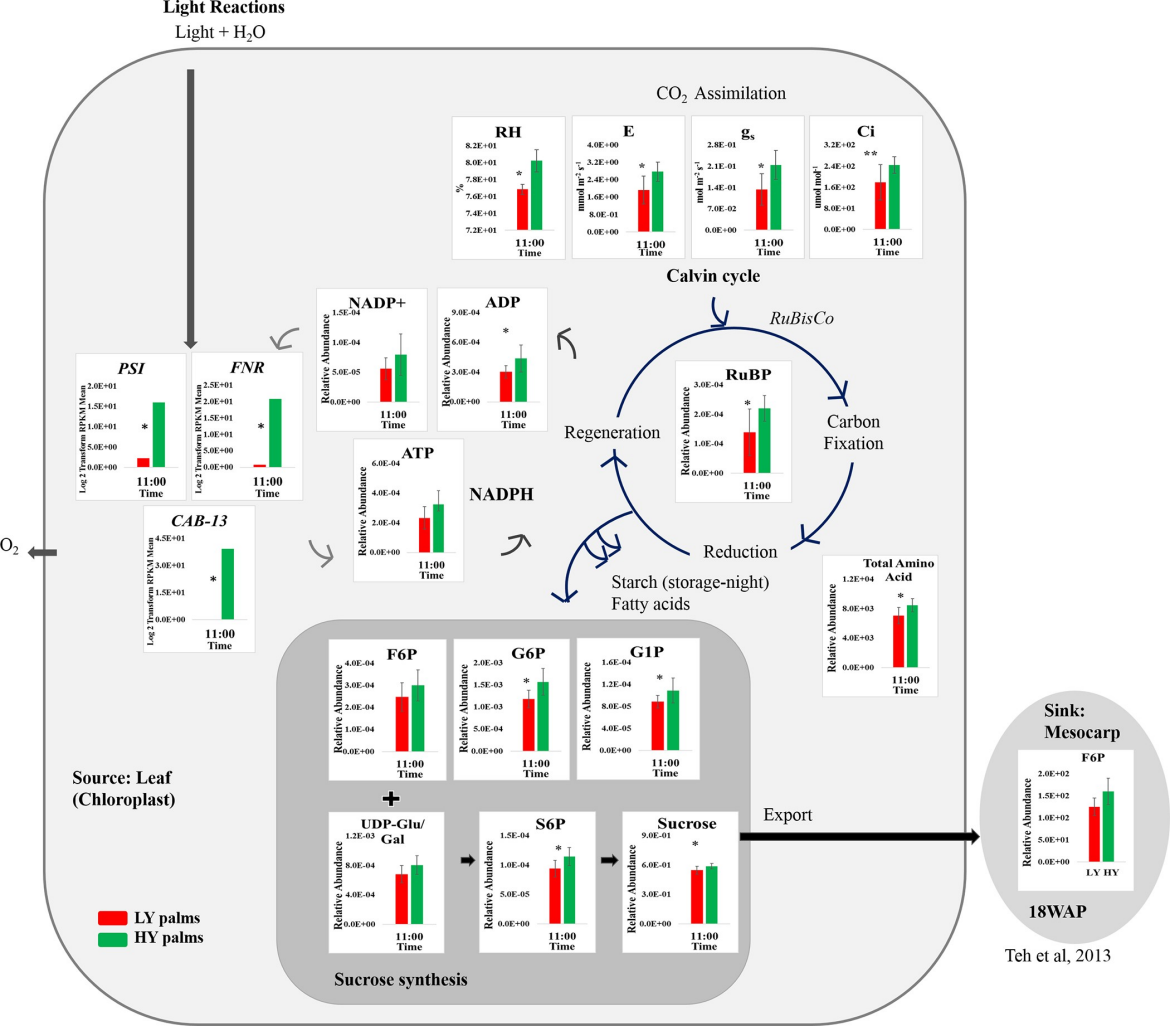
Differential photosynthesis gene expressions, gas exchange and the Calvin cycle in HY and LY oil palm leaves. Green bar represents the average of HY (n = 5 for gas exchange; n = 10 for metabolites and expressions); Red bar—the average of LY (n = 5 for gas exchange; n = 10 for metabolites and expressions) collected in the open field. * represent a significant difference between HY and LY with *p*-value ≤ 0.05; The concentration of metabolites was quantified by abundance relative to the internal standard, Ribitol (2 mg mL^-1^).

Comparison of gene expression and metabolite concentrations throughout the 24-hour diurnal cycle identified three important photosynthesis genes associated with HY oil palms, particularly under low light. As shown in Fig 6, the high concentration of sugars in HY palms after the period of low light (11:00) was supported by analysis of gene expression where remarkable differences were noted in key photosynthesis-related genes at this time. Significantly higher expression of *CAB-13* (10.3-fold), *PSI* (3.3-fold) and *FNR* (5.6-fold) were observed in the HY group over their expression in LY at 11:00. However, immediately after the period of high light intensity (15:00), the expressions of these photosynthetic genes in HY palm, while still higher, showed much less differential (1.6 to 2.8-fold higher than LY). According to Gibon et al., changes in metabolite concentrations follow the enzyme activities involved in central carbon and nitrogen metabolism, and changes in concentration are delayed compared to changes in transcript levels, particularly after darkness, and varied from enzyme to enzyme [15]. In agreement with this, the concentrations of sugars were observed to continue to increase to a maximum at the end of the peak midday light intensity (15:00) and then decrease after that (Table 1).

Light harvesting *CAB-13* is one of the most abundant proteins in the chloroplast, with the main function of collecting and transferring light energy to photosynthesis reaction centers. In *Arabidopsis thaliana*, genetic evidence showed the type III light-harvesting *CAB-13* gene to be a major factor responsible for light tolerance [32]. Introgression of the tolerance into modern tomato hybrid lines resulted in a yield increment of up to 20%, although continuous light-tolerant leaves only showed a slight decrease in the rate of photosynthesis across light and CO_2_ levels. According to Velez-Ramirez, this protein was thought to have a regulatory role in balancing light harvesting by *PSI* and *photosystems II (PSII)*[32]. Single nucleotide polymorphisms (SNPs) found in the *CAB* in barley have been found to be associated with at least one of six agronomic traits: plant height, spike length, number of grains per spike, thousand grain weight, flag leaf area and leaf color [33]. Comparison of differentially expressed genes in seeds of two *Brassica napus* lines revealed the importance of *CAB* as a novel target for genetic improvement of seed oil [34]. The microarray analysis of gene expression in *Brassica napus* at different location and altitude of China also identified several family genes of *CAB*. The author suggested that upregulation of these genes in higher oil content *Brassica napus* seeds could improve photosynthesis and eventually improved fatty acid synthesis [35]. The exploration of genetic variation in *CAB-13* genes provides useful selection tools as potential markers to improve plants through higher photosynthetic capacity.

PSI is a multisubunit complex membrane-bound protein in plants that use light energy to mediate electron transfer from plastocyanin to FNR. It comprises two closely related protein subunits, namely PsaA and PsaB, that bind to P700 as the primary electron donor in photosynthesis. The differential in the expression of *PSI reaction center subunit XI* (isotig69597) was observed during 7:00 and 19:00 throughout the diurnal cycle.

Ferredoxin (FNR) is an iron-sulfur protein that is involved in electron transfer in a wide range of metabolic reactions. Double knockout of the *Arabidopsis FNR* genes prevented the autotrophic development of the mutant plants, resulting in a small and pale phenotype of the plant with down-regulated photosynthetic capacity [36–41]. Our transcriptome analysis showed significantly higher expression of *FNR* in HY than in LY oil palms under both low and high light intensity.

The observation of differential photosynthetic gene expression was further supported by analysis of gas exchange in the plants studied. Unlike for small plants that can be assessed in a growth chamber, photosynthetic studies of mature oil palm must be conducted in actual plantation fields where the average plant height ranges from two meters to greater than eight meters. This precludes concurrent measurements of many palms using portable photosynthesis instruments, such as LICOR. Hence, in this study, gas exchange measurements were conducted by using LICOR on only five palms each from HY and LY due to sampling constraints. In Malaysia, oil palm g_s_ has been reported to peak mid-morning and then progressively decline in the afternoon [42, 43]. However, our current findings indicated that g_s_ and E decreased from sunrise (07:00) to mid- morning (11:00) and increased in the afternoon (15:00), (Fig 4). According to Gomez et al., [44] who cited Hernandex et al., [45], VPD could negatively affect g_s_ [46, 47] and CO_2_ assimilation due to stomatal closure. The diurnal variation of oil palm Ci was similar to coffee leaves [44], where it decreased from sunrise to mid- morning due to an increase of A. Ci was then increased after 11:00 as A decreased, indicating the coupling of A and Ci. According to Long et al., [48] leaf A did not correlate well with yield when different genotypes of a crop species are compared, but elevated CO_2_ does. In this present study under a similar environment of ambient CO_2_, variation was noted in the transpiration and stomatal activity. In comparison to the CO_2_ assimilation between HY and LY, the level of Ci, E and g_s_ were higher in HY where the *p-*value ≤ 0.10 at 07:00 and 11:00. In oil palm under the ambient condition in the field, Ci of HY palm was found to be 35% higher compared to LY palm, indicating the potential of higher CO_2_ assimilation in HY palm. In the HY palms, we also observed a 60% higher of *g*_s_ compared to LY. According to a study on C3 plant, high *g*_s_ rates are critical to maximizing yield particular during seed formation and filling [49]. In an earlier review by Zelitch [50], researchers have shown that improving photosynthetic efficiency through photosynthetic energy transduction and CO_2_ assimilation in crops may help to increase crop yield significantly. On average, a doubling of the current CO_2_ concentration in the field or laboratory chambers results in no increase in leaf area, but can increase an average of 35% in crop yield [51]. Even though the diurnal changes of A and T were obvious, but there were no differences between HY and LY. This might due to the spatial variation in gas exchange where g_s_ and A are not consistently well correlated in different area [52]. Given the nature of spatial variation and rapid changes in A, g_s_, E and Ci throughout the day, it is not trivial to sample statistically relevant groups of palms efficiently and accurately with reasonable resources.

One of the main factors influencing electron transfer in the chloroplast is the efficiency of CO_2_ fixation and of energy conversion during the high light intensity period, which can be associated with the potential rate of CO_2_ fixation by ribulose-1,5-bisphosphate carboxylase/oxygenase (RuBisCo). The fixation rate is determined by the amount of RuBisCo present and its kinetic properties as well as the capacity of chloroplast stromal reaction to regenerate ribulose-1,5-bisphosphate (RuBP), which has been found to be a substrate for the RuBisCo reaction [8]. In the present study, a higher RuBP concentration was observed in HY palm with significant differences at 11:00 and 15:00 (*p*-value ≤ 0.05) (Fig 2A).

In the reduction stage of the Calvin cycle, NADPH and ATP actively reduce triose phosphate to hexose phosphates (G1P, G6P, and F6P) where the sugar biosynthesis begins. With the presence of uridine diphosphates-glucose (UDP-glu), S6P is produced and then converted to sucrose as the end product. The diurnal changes were more profound for sugars such as G6P, G1P, UDP-glu/gal, S6P and sucrose in both HY and LY groups, especially under low light (*p*-value ≤ 0.05). Low sugar levels during low light have been proposed to be the main factor in diurnal gene regulation [14] and may be a relative limiter for LY palms. Under low light again, at 19:00, the *p*-values for sugars and sugar phosphates ranges between HY and LY (0.014 to 0.17) probably due to exhaustion of sugars assimilation from leaves to sink. The higher concentration of sucrose, sugar phosphates and nucleotides in HY palms suggests a better production and turnover of sugars from photosynthesis under low light. The higher concentration of sucrose in HY palms has been verified twice in two palm populations [53].

The differential expression of key photosynthesis genes, including *CAB*, *PSI*, and *FNR*, under low light intensity may indicate that HY oil palms are more efficient at energy capture from sunlight through *CAB*. The captured energy is then channeled more efficiently through electron transfer by *FNR*, ultimately resulting in faster accumulation of photosynthate (sugars and amino acids). This photosynthate is then available for transport to other parts of the plant and ultimately for consumption by the rapidly developing fruit.

A snapshot of short-term cellular processes from photosynthetic leaf sources leading to the long-term process of mesocarp oil accumulation in the sink is showed in Fig 6. The glycolytic pathway is suggested to play an important role in the development of oil palm fruits in HY palms. Lipid formation in oil palm mesocarp starts as early as 16 WAP, reaching maximum at around 22 WAP [54]. Immediately prior to oil biosynthesis (14 to 16 WAP), total sugars in oil palm mesocarp have been found to be more abundant in high-yield palms, leading to a concomitant higher increment of lipid content from 18 WAP onwards [26]. The early intermediates F6P were also observed to have higher concentrations in high yield palms at the early stages of lipid biosynthesis. This provides further support for a hypothesis of higher sugar production in leaves eventually resulting in higher oil biosynthesis in the fruit during ripening.

## Conclusions

In this study, the comparison of leaf gas exchange and biochemical observations throughout a diurnal cycle for oil palm groups revealed the significantly faster accumulation of sugar phosphates in HY palms during the period of low light in the morning (between 07:00 and 11:00). This observation was further supported by transcriptome analysis in which 191 DEGs were identified including three photosynthetic genes namely *CAB-13*, *PSI* and *FNR*. All of these genes were found to be significantly expressed in HY palms and could serve as potential biomarkers for the selection of improved planting materials, particularly for conditions of limited light. However, in addition to these, many other DEGs identified were unannotated and would need further validation before being adopted as biomarkers. This discovery of differential photosynthetic machinery and metabolites under low light has important implications for future selection of plants that perform better at peak maturity and with high planting density where light competition may result in yield limitation. In conjunction with the sustainability aim to increase yield per unit of land area, selection of planting materials via photosynthesis-related biomarkers for high-density planting is definitely of significant interest to boost plantation productivity without the need for more land.

## Supporting information

S1 Fig Expression comparison of 3 photosynthesis genes between high yielding (HY) and low yielding (LY) at different light conditions using qPCR. (TIF)

S2 Fig **Comparison of metabolites differences between HY and LY using PCA for A) All time points, B) 07:00, C)11:00, D) 15:00, E) 19:00, and F) Day 2 07:00**.
(TIF)

S3 Fig **Comparison of metabolites differences between HY and LY using OPLS and S-plot analysis for A) All time points, B) 07:00, C)11:00, D) 15:00, E) 19:00, and F) Day 2 07:00**.
(TIF)

S1 Table Oil palm bunch analysis and vegetative measurement. (DOCX)

S2 Table Metabolites concentration of oil palm leaves (L12 and L17) sampled in three different time points (one month laps). (DOCX)

S3 Table Metabolites concentration comparison of HY and LY palms. (DOCX)

S4 Table Total starch content comparison of HY and LY palms. (DOCX)

S5 Table Differentially expressed genes list for individual diurnal time point from transcriptome sequencing. (DOCX)

S6 Table The annotation of the transcript/s within a cluster. (DOCX)

S7 Table Gene Ontology enrichment analysis of individual diurnal time point. (DOCX)
