# Supplementary figures and images for: Diurnal biomarkers reveal key photosynthetic genes associated with increased oil palm yield
Source: PLoS One. 2019 Mar 11;14(3):e0213591. doi: 10.1371/journal.pone.0213591 (PMC6411157; doi:10.1371/journal.pone.0213591)

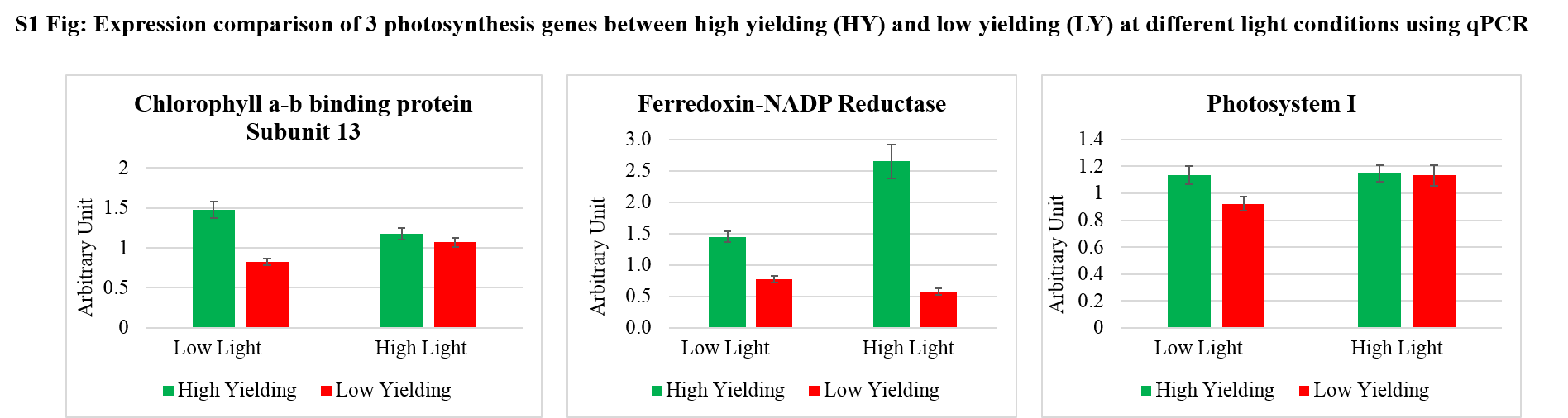

Supplement: S1 Fig — (TIF) [file pone.0213591.s001.tif]

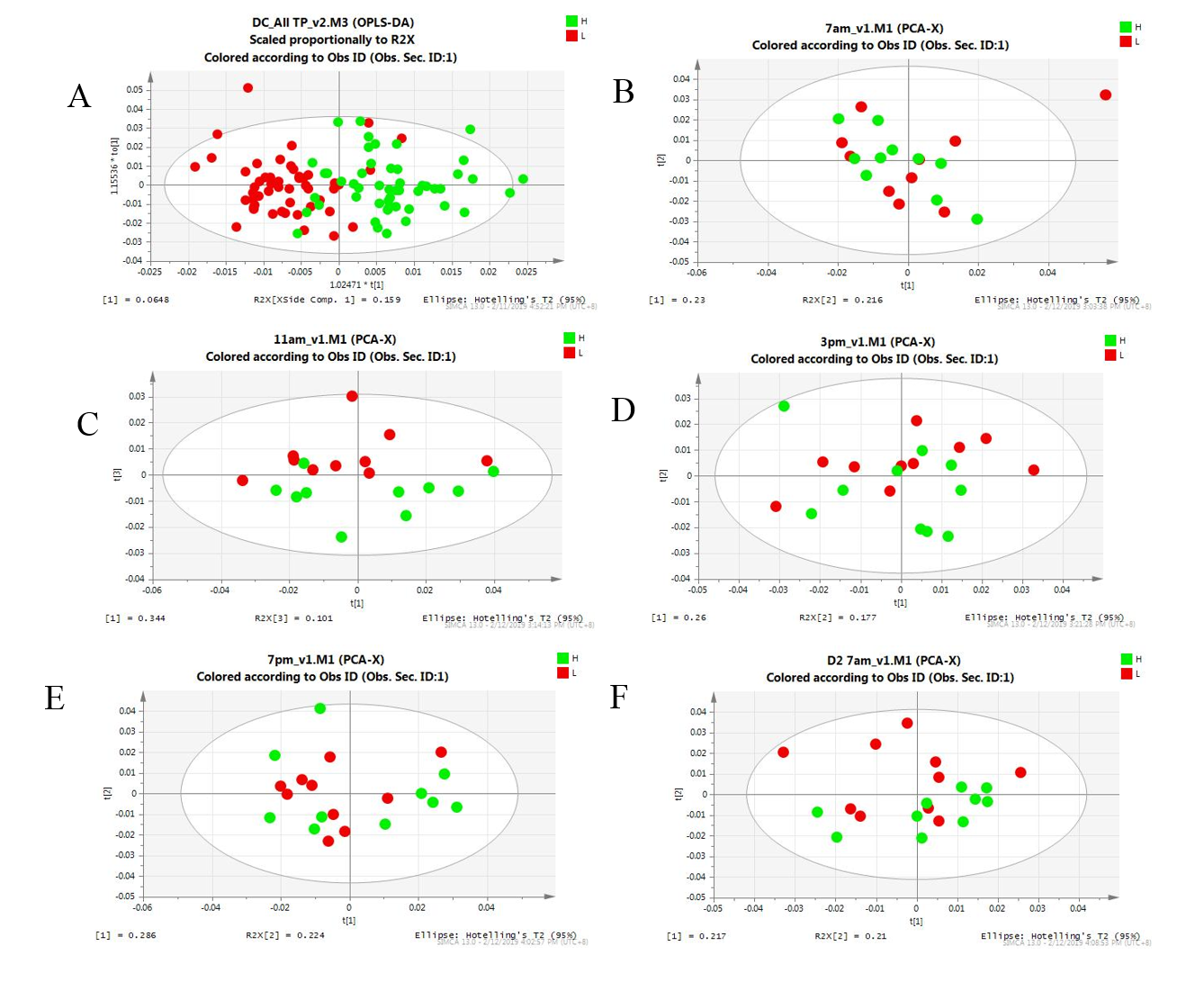

Supplement: S2 Fig — Comparison of metabolites differences between HY and LY using PCA for A) All time points, B) 07:00, C)11:00, D) 15:00, E) 19:00, and F) Day 2 07:00. (TIF) [file pone.0213591.s002.tif]

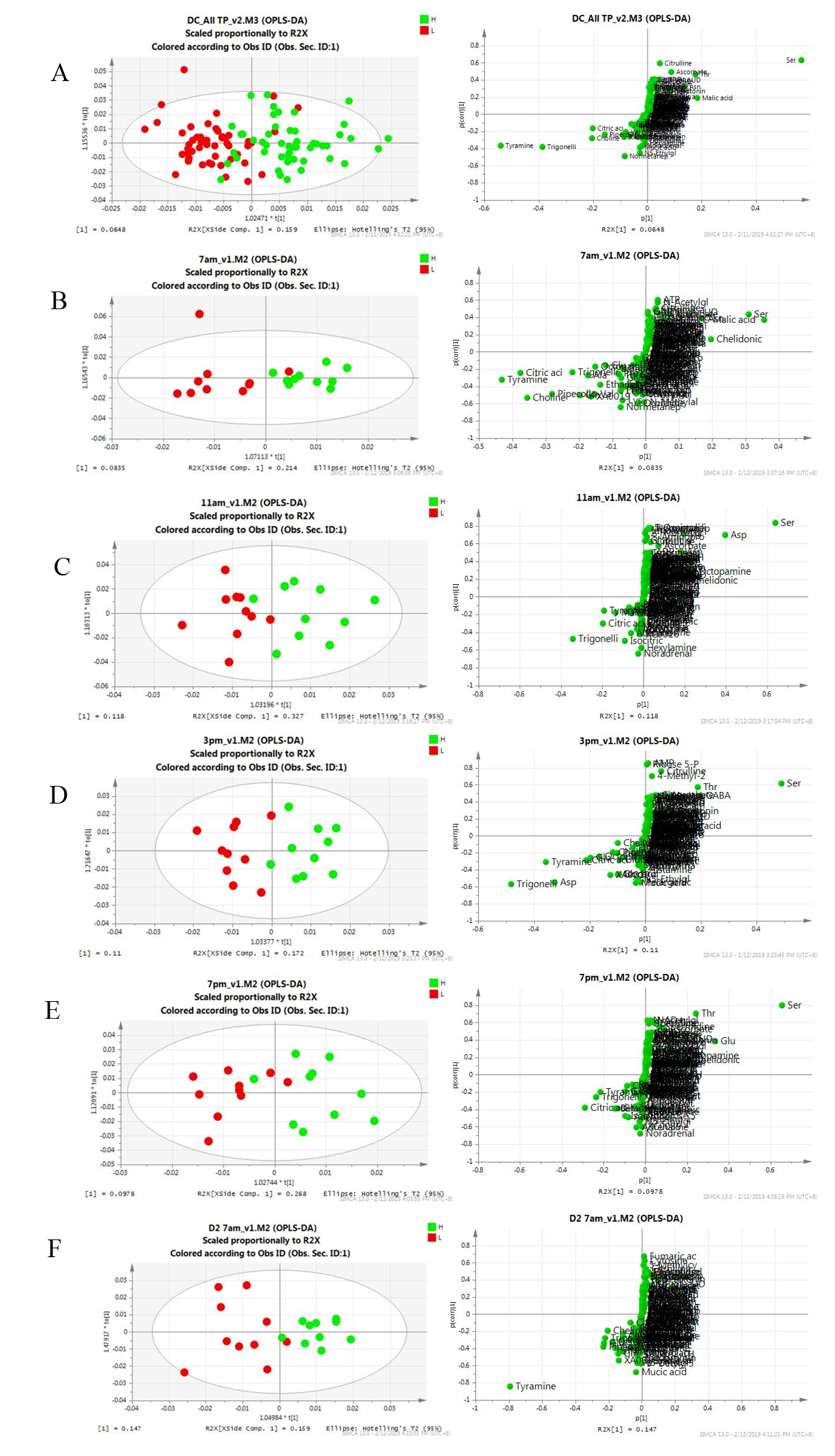

Supplement: S3 Fig — Comparison of metabolites differences between HY and LY using OPLS and S-plot analysis for A) All time points, B) 07:00, C)11:00, D) 15:00, E) 19:00, and F) Day 2 07:00. (TIF) [file pone.0213591.s003.tif]
