# Supplementary material for: Diurnal biomarkers reveal key photosynthetic genes associated with increased oil palm yield
Source: PLoS One. 2019 Mar 11;14(3):e0213591. doi: 10.1371/journal.pone.0213591 (PMC6411157; doi:10.1371/journal.pone.0213591)
Supplement: S1 Table — (DOCX) [file pone.0213591.s004.docx]

| **Supplementary table 1** | | | |  |  |  |  |  |  |  |  |  |  |  |  |  |
| --- | --- | --- | --- | --- | --- | --- | --- | --- | --- | --- | --- | --- | --- | --- | --- | --- |
| Oil Palm Bunch Analysis and Vegetative Measurement | | | | | | |  |  |  |  |  |  |  |  |  |  |
|  |  |  |  |  |  |  |  |  |  |  |  |  |  |  |  |  |
|  | Fresh fruit bunch | | Bunch Number | Bunch Weight | Number of Fruitlets per Bunch | Fresh Mesocarp Weight | Mesocarp per Fruit | Shell per Fruit | Kernel per Fruit | Oil in Dry Mesocarp | Oil in Wet Mesocarp | Oil per Bunch | Kernel per Bunch | Oil per Palm | Oil per hectare |  |
|  | (t/h/yr) | (kg/p/yr) | (No/p/yr) | (kg) | (%) | (g) | (%) | (%) | (%) | (%) | (%) | (%) | (%) | (kg/p/yr) | (t/h/yr) |  |
| Average_LY | 22.9 | 168.6 | 20.3 | 8.4 | 58.9 | 11.4 | 78.4 | 12.1 | 9.5 | 74.7 | 49.6 | 23.0 | 5.1 | 38.7 | 5.3 |  |
| SD_LY | 3.94 | 28.99 | 3.57 | 0.82 | 5.88 | 2.00 | 6.72 | 3.74 | 3.06 | 2.17 | 1.77 | 3.49 | 1.86 | 8.75 | 1.19 |  |
| Average_HY | 22.7 | 166.8 | 18.6 | 9.0 | 61.7 | 14.1 | 85.6 | 8.4 | 6.1 | 79.3 | 55.0 | 29.1 | 2.8 | 48.5 | 6.6 |  |
| SD_HY | 2.45 | 18.00 | 2.40 | 0.78 | 6.56 | 1.34 | 4.97 | 2.65 | 2.43 | 0.93 | 1.33 | 4.18 | 1.16 | 8.08 | 1.10 |  |
| *p*-value | 0.87 | 0.87 | 0.22 | 0.07 | 0.32 | 0.00 | 0.01 | 0.02 | 0.01 | 0.00 | 0.00 | 0.00 | 0.00 | 0.02 | 0.02 |  |
|  |  |  |  |  |  |  |  |  |  |  |  |  |  |  |  |  |
|  |  |  |  |  |  |  |  |  |  |  |  |  |  |  |  |  |
|  |  |  |  |  |  |  |  |  |  |  |  |  |  |  |  |  |
|  | Frond length | Rachis Weight | Frond Weight | Number of Pinnae | Left Middle Pinnae | | Right Middle Pinnae | | Rachis Width | Rachis |  |  |  |  |  |  |
|  | (cm) | (g) | (g) |  | Length (cm) | Width (cm) | Length (cm) | Width (cm) | (cm) | (cm) |  |  |  |  |  |  |
| Average_LY | 517.7 | 3180.0 | 5440.0 | 297.4 | 95.3 | 5.1 | 97.0 | 5.3 | 7.6 | 3.9 |  |  |  |  |  |  |
| SD_LY | 38.38 | 829.73 | 1097.67 | 25.73 | 7.89 | 0.48 | 8.28 | 0.51 | 0.98 | 0.50 |  |  |  |  |  |  |
| Average_HY | 522.1 | 2955.6 | 5144.4 | 291.1 | 93.9 | 5.4 | 95.2 | 5.6 | 7.7 | 3.9 |  |  |  |  |  |  |
| SD_HY | 35.45 | 512.62 | 712.59 | 23.24 | 7.08 | 0.50 | 7.09 | 0.53 | 0.55 | 0.28 |  |  |  |  |  |  |
| *p*-value | 0.80 | 0.49 | 0.50 | 0.59 | 0.70 | 0.29 | 0.63 | 0.29 | 0.86 | 0.97 |  |  |  |  |  |  |
|  |  |  |  |  |  |  |  |  |  |  |  |  |  |  |  |  |
| LY | Low mesocarp oil content | | | |  |  |  |  |  |  |  |  |  |  |  |  |
| HY | High mesocarp oil content | | | |  |  |  |  |  |  |  |  |  |  |  |  |
|  |  |  |  |  |  |  |  |  |  |  |  |  |  |  |  |  |
