# Supplementary material for: Diurnal biomarkers reveal key photosynthetic genes associated with increased oil palm yield
Source: PLoS One. 2019 Mar 11;14(3):e0213591. doi: 10.1371/journal.pone.0213591 (PMC6411157; doi:10.1371/journal.pone.0213591)
Supplement: S2 Table — (DOCX) [file pone.0213591.s005.docx]

**Supplementary table 2**

Metabolites concentration of oil palm leaves (L12 and L17) sampled in three different time points (one month laps)

|  | Palm 1 | | | | | |  | Palm 2 | | | | | |  | Palm 1 | |  | Palm 2 | | |
| --- | --- | --- | --- | --- | --- | --- | --- | --- | --- | --- | --- | --- | --- | --- | --- | --- | --- | --- | --- | --- |
|  | T1_12 | T2_12 | T3_12 | T1_17 | T2_17 | T3_17 |  | T1_12 | T2_12 | T3_12 | T1_17 | T2_17 | T3_17 |  | Frond 12 | Frond  17 | | Frond 12 | | Frond  17 |
|  |  |  |  |  |  |  |  |  |  |  |  |  |  |  |  |  |  |  |  | |
| Ala | 0.026 | 0.031 | 0.019 | 0.025 | 0.021 | 0.019 |  | 0.048 | 0.035 | 0.030 | 0.021 | 0.038 | 0.031 |  | 0.025 | 0.022 |  | 0.038 | 0.030 | |
| Asn | 0.059 | 0.063 | 0.085 | 0.049 | 0.057 | 0.071 |  | 0.103 | 0.061 | 0.075 | 0.099 | 0.051 | 0.064 |  | 0.069 | 0.059 |  | 0.080 | 0.071 | |
| Arg | 0.698 | 0.800 | 0.523 | 0.443 | 0.550 | 0.639 |  | 0.609 | 0.566 | 0.444 | 0.797 | 0.585 | 0.517 |  | 0.674 | 0.544 |  | 0.540 | 0.633 | |
| Asp | 0.366 | 0.370 | 0.551 | 0.276 | 0.335 | 0.455 |  | 0.609 | 0.354 | 0.443 | 0.632 | 0.277 | 0.403 |  | 0.429 | 0.355 |  | 0.469 | 0.437 | |
| Citruline | 0.063 | 0.088 | 0.067 | 0.060 | 0.063 | 0.058 |  | 0.093 | 0.056 | 0.047 | 0.064 | 0.062 | 0.049 |  | 0.072 | 0.060 |  | 0.065 | 0.058 | |
| Cys | 0.000 | 0.000 | 0.000 | 0.000 | 0.000 | 0.000 |  | 0.000 | 0.000 | 0.000 | 0.000 | 0.000 | 0.000 |  | 0.000 | 0.000 |  | 0.000 | 0.000 | |
| GABA | 0.217 | 0.204 | 0.178 | 0.235 | 0.135 | 0.101 |  | 0.439 | 0.093 | 0.097 | 0.091 | 0.246 | 0.165 |  | 0.200 | 0.157 |  | 0.209 | 0.167 | |
| Gln | 0.406 | 0.583 | 0.266 | 0.346 | 0.584 | 0.295 |  | 1.002 | 0.486 | 0.377 | 0.400 | 0.652 | 0.380 |  | 0.418 | 0.409 |  | 0.622 | 0.477 | |
| Glu | 2.722 | 3.228 | 2.325 | 5.047 | 5.170 | 2.184 |  | 4.688 | 3.204 | 3.759 | 4.679 | 5.097 | 3.131 |  | 2.758 | 4.134 |  | 3.884 | 4.302 | |
| Gly | 0.000 | 0.000 | 0.000 | 0.000 | 0.000 | 0.000 |  | 0.000 | 0.000 | 0.000 | 0.000 | 0.000 | 0.000 |  | 0.000 | 0.000 |  | 0.000 | 0.000 | |
| His | 0.608 | 0.751 | 0.691 | 0.812 | 0.708 | 0.688 |  | 0.743 | 0.862 | 0.657 | 0.849 | 0.910 | 0.716 |  | 0.683 | 0.736 |  | 0.754 | 0.825 | |
| Homoserine | 0.143 | 0.201 | 0.150 | 0.144 | 0.163 | 0.141 |  | 0.268 | 0.210 | 0.163 | 0.181 | 0.247 | 0.157 |  | 0.165 | 0.149 |  | 0.214 | 0.195 | |
| Hydroxyproline | 0.002 | 0.003 | 0.002 | 0.002 | 0.002 | 0.001 |  | 0.003 | 0.002 | 0.001 | 0.001 | 0.003 | 0.001 |  | 0.002 | 0.002 |  | 0.002 | 0.001 | |
| Leu/Ile | 0.217 | 0.274 | 0.243 | 0.354 | 0.313 | 0.155 |  | 0.410 | 0.473 | 0.250 | 0.209 | 0.540 | 0.229 |  | 0.245 | 0.274 |  | 0.378 | 0.326 | |
| Lys | 0.907 | 1.096 | 0.583 | 0.758 | 1.225 | 0.675 |  | 2.154 | 0.951 | 0.765 | 0.760 | 1.230 | 0.770 |  | 0.862 | 0.886 |  | 1.290 | 0.920 | |
| Met | 0.001 | 0.001 | 0.001 | 0.001 | 0.001 | 0.000 |  | 0.002 | 0.001 | 0.001 | 0.001 | 0.001 | 0.001 |  | 0.001 | 0.001 |  | 0.001 | 0.001 | |
| Orn |  | 0.002 | 0.007 | 0.002 | 0.003 | 0.003 |  | 0.007 | 0.003 | 0.002 | 0.006 | 0.004 |  |  | 0.004 | 0.002 |  | 0.004 | 0.005 | |
| Phe | 0.018 | 0.031 | 0.022 | 0.027 | 0.029 | 0.015 |  | 0.037 | 0.051 | 0.026 | 0.022 | 0.065 | 0.021 |  | 0.024 | 0.023 |  | 0.038 | 0.036 | |
| Pro | 0.034 | 0.043 | 0.034 | 0.033 | 0.032 | 0.032 |  | 0.044 | 0.042 | 0.035 | 0.028 | 0.039 | 0.035 |  | 0.037 | 0.033 |  | 0.040 | 0.034 | |
| Tyr | 0.022 | 0.024 | 0.020 | 0.033 | 0.033 | 0.017 |  | 0.021 | 0.033 | 0.022 | 0.026 | 0.047 | 0.019 |  | 0.022 | 0.027 |  | 0.025 | 0.031 | |
| Trp | 1.535 | 1.691 | 2.001 | 6.659 | 4.438 | 0.686 |  | 2.837 | 2.662 | 2.208 | 12.901 | 5.985 | 2.747 |  | 1.742 | 3.928 |  | 2.569 | 7.211 | |
| Val | 0.578 | 0.781 | 0.535 | 0.568 | 0.658 | 0.425 |  | 0.905 | 0.830 | 0.456 | 0.321 | 0.849 | 0.427 |  | 0.631 | 0.551 |  | 0.730 | 0.532 | |
| Spermidine | 0.425 | 0.778 | 0.791 | 1.102 | 0.796 | 0.789 |  | 0.470 | 0.808 | 0.494 | 0.661 | 0.674 | 0.898 |  | 0.665 | 0.896 |  | 0.591 | 0.744 | |
| Spermine | 0.103 | 0.143 | 0.149 | 0.212 | 0.187 | 0.172 |  | 0.267 | 0.166 | 0.091 | 0.128 | 0.094 | 0.154 |  | 0.132 | 0.190 |  | 0.174 | 0.125 | |
| Adenine | 0.014 | 0.014 | 0.012 | 0.023 | 0.020 | 0.008 |  | 0.011 | 0.019 | 0.012 | 0.014 | 0.029 | 0.011 |  | 0.013 | 0.017 |  | 0.014 | 0.018 | |
| Cytosine | 7.128 | 8.777 | 7.782 | 5.867 | 7.366 | 7.576 |  | 8.308 | 7.902 | 6.482 | 5.450 | 5.512 | 6.862 |  | 7.896 | 6.936 |  | 7.564 | 5.941 | |
| Guanine | 13.327 | 14.975 | 13.554 | 14.234 | 14.463 | 14.605 |  | 14.443 | 15.364 | 13.322 | 13.484 | 13.995 | 14.103 |  | 13.952 | 14.434 |  | 14.376 | 13.860 | |
| Thymine | 9.385 | 10.551 | 9.217 | 7.150 | 8.342 | 8.722 |  | 10.959 | 10.670 | 9.808 | 9.660 | 8.774 | 9.956 |  | 9.718 | 8.072 |  | 10.479 | 9.463 | |
| Uracil | 0.003 | 0.007 | 0.006 | 0.003 | 0.002 | 0.002 |  | 0.008 | 0.002 | 0.001 | 0.003 | 0.002 | 0.002 |  | 0.005 | 0.003 |  | 0.004 | 0.002 | |
| Uridine | 0.001 | 0.004 | 0.003 | 0.034 | 0.012 | 0.001 |  | 0.006 | 0.006 | 0.008 | 0.014 | 0.021 | 0.005 |  | 0.003 | 0.016 |  | 0.007 | 0.013 | |
| Carnosine | 0.004 | 0.004 | 0.005 | 0.003 | 0.004 | 0.004 |  | 0.003 | 0.005 | 0.002 | 0.003 | 0.003 | 0.004 |  | 0.004 | 0.004 |  | 0.003 | 0.003 | |
| Caffeic acid | 0.005 | 0.005 | 0.005 | 0.007 | 0.006 | 0.005 |  | 0.005 | 0.006 | 0.005 | 0.007 | 0.007 | 0.006 |  | 0.005 | 0.006 |  | 0.005 | 0.007 | |
| Anthranilate | 0.013 | 0.035 | 0.027 | 0.037 | 0.041 | 0.019 |  | 0.056 | 0.038 | 0.040 | 0.043 | 0.045 | 0.025 |  | 0.025 | 0.032 |  | 0.045 | 0.038 | |
| Creatine | 0.006 | 0.013 | 0.012 | 0.016 | 0.016 | 0.011 |  | 0.013 | 0.011 | 0.012 | 0.013 | 0.016 | 0.030 |  | 0.010 | 0.015 |  | 0.012 | 0.020 | |
| S-adenosyl methionine | 0.019 | 0.025 | 0.024 | 0.025 | 0.023 | 0.018 |  | 0.032 | 0.034 | 0.031 | 0.027 | 0.043 | 0.030 |  | 0.023 | 0.022 |  | 0.033 | 0.033 | |
| Tyramine | 0.460 | 0.343 | 0.174 | 0.047 | 0.189 | 0.670 |  | 0.141 | 0.269 | 0.142 | 0.050 | 0.063 | 0.171 |  | 0.326 | 0.302 |  | 0.184 | 0.095 | |
| Hypoxanthine | 0.062 | 0.062 | 0.035 | 0.039 | 0.067 | 0.048 |  | 0.049 | 0.075 | 0.056 | 0.040 | 0.064 | 0.058 |  | 0.053 | 0.051 |  | 0.060 | 0.054 | |
| 3-phosphoglyceric acid | 0.305 | 0.259 | 0.286 | 0.252 | 0.404 | 0.370 |  | 0.243 | 0.214 | 0.292 | 0.241 | 0.267 | 0.249 |  | 0.284 | 0.342 |  | 0.249 | 0.252 | |
| 5-phosphoribosyl-1-pyrophosphate | 0.000 | 0.000 | 0.000 | 0.001 | 0.000 | 0.001 |  | 0.001 | 0.000 | 0.001 | 0.000 | 0.000 | 0.001 |  | 0.000 | 0.001 |  | 0.000 | 0.001 | |
| 6-phosphogluconic acid | 0.004 | 0.001 |  | 0.001 | 0.001 |  |  | 0.001 | 0.002 | 0.000 | 0.002 | 0.000 | 0.001 |  | 0.002 | 0.001 |  | 0.001 | 0.001 | |
| Dihydroxyacetone phosphate | 0.011 | 0.008 | 0.007 | 0.007 | 0.006 | 0.005 |  | 0.012 | 0.006 | 0.014 | 0.007 | 0.010 | 0.008 |  | 0.008 | 0.006 |  | 0.010 | 0.009 | |
| Erythrose-4-phosphate | 0.052 | 0.055 | 0.049 | 0.039 | 0.077 | 0.044 |  | 0.051 | 0.035 | 0.056 | 0.068 | 0.034 | 0.045 |  | 0.052 | 0.054 |  | 0.047 | 0.049 | |
| Fructose-6-phosphate | 2.295 | 2.408 | 2.147 | 2.068 | 3.203 | 2.234 |  | 2.698 | 1.816 | 2.996 | 3.331 | 1.794 | 2.033 |  | 2.283 | 2.502 |  | 2.503 | 2.386 | |
| Fructose-1,6-phoshate | 0.006 | 0.006 | 0.005 | 0.006 | 0.008 | 0.011 |  | 0.007 | 0.006 | 0.006 | 0.005 | 0.006 | 0.005 |  | 0.006 | 0.008 |  | 0.006 | 0.005 | |
| Glucose-1-phospahate/ Glucose-6-phosphate | 0.021 | 0.026 | 0.024 | 0.021 | 0.044 | 0.022 |  | 0.027 | 0.024 | 0.029 | 0.030 | 0.021 | 0.019 |  | 0.024 | 0.029 |  | 0.027 | 0.024 | |
| Glycerol-3-phosphate | 0.239 | 0.214 | 0.142 | 0.186 | 0.225 | 0.147 |  | 0.309 | 0.208 | 0.196 | 0.170 | 0.231 | 0.199 |  | 0.198 | 0.186 |  | 0.238 | 0.200 | |
| Glyceraldehyde-3-phosphate | 0.009 | 0.009 | 0.007 | 0.004 | 0.006 | 0.006 |  | 0.014 | 0.006 | 0.013 | 0.009 | 0.007 | 0.009 |  | 0.008 | 0.006 |  | 0.011 | 0.008 | |
| Phosphoenolpyruvic acid | 0.001 | 0.001 | 0.001 | 0.000 | 0.001 | 0.001 |  | 0.000 | 0.001 | 0.002 | 0.001 | 0.002 | 0.001 |  | 0.001 | 0.001 |  | 0.001 | 0.001 | |
| Ribose-5-phosphate | 0.086 | 0.077 | 0.054 | 0.069 | 0.116 | 0.053 |  | 0.099 | 0.083 | 0.083 | 0.072 | 0.087 | 0.064 |  | 0.072 | 0.079 |  | 0.088 | 0.074 | |
| Ribulose-5-phosphate | 0.079 | 0.075 | 0.047 | 0.067 | 0.109 | 0.051 |  | 0.101 | 0.072 | 0.082 | 0.077 | 0.077 | 0.057 |  | 0.067 | 0.076 |  | 0.085 | 0.071 | |
| Xylulose-5-phosphate | 0.029 | 0.030 | 0.021 | 0.024 | 0.048 | 0.024 |  | 0.032 | 0.031 | 0.034 | 0.028 | 0.030 | 0.028 |  | 0.026 | 0.032 |  | 0.032 | 0.028 | |
| Glutathione (red) | 0.005 | 0.007 | 0.008 | 0.005 | 0.007 | 0.003 |  | 0.088 | 0.009 | 0.024 | 0.010 | 0.012 | 0.010 |  | 0.007 | 0.005 |  | 0.041 | 0.011 | |
| Glutathione (ox) | 0.186 | 0.154 | 0.236 | 0.121 | 0.154 | 0.184 |  | 0.899 | 0.233 | 0.480 | 0.170 | 0.367 | 0.329 |  | 0.192 | 0.153 |  | 0.538 | 0.289 | |
| Dehydroshikimic acid | 0.021 | 0.018 | 0.015 | 0.053 | 0.041 | 0.013 |  | 0.042 | 0.023 | 0.040 | 0.032 | 0.029 | 0.024 |  | 0.018 | 0.036 |  | 0.035 | 0.028 | |
| 2-Oxoglutaric acid | 0.067 | 0.036 | 0.017 | 0.104 | 0.039 | 0.014 |  | 0.012 | 0.240 | 0.033 | 0.065 | 0.045 | 0.029 |  | 0.040 | 0.052 |  | 0.095 | 0.046 | |
| Isocitric acid | 1.538 | 1.172 | 0.823 | 7.474 | 1.324 | 1.016 |  | 0.625 | 5.671 | 1.572 | 1.802 | 2.041 | 1.355 |  | 1.178 | 3.271 |  | 2.623 | 1.732 | |
| Malic acid | 6.843 | 6.405 | 2.792 | 33.193 | 6.031 | 3.559 |  | 3.555 | 26.576 | 6.868 | 8.506 | 10.030 | 6.641 |  | 5.347 | 14.261 |  | 12.333 | 8.392 | |
| Glycolic acid | 0.002 | 0.002 | 0.001 | 0.005 | 0.001 | 0.000 |  | 0.001 | 0.002 | 0.003 | 0.001 | 0.002 | 0.002 |  | 0.001 | 0.002 |  | 0.002 | 0.002 | |
| Lactic acid | 0.000 | 0.000 | 0.001 | 0.002 | 0.001 | 0.000 |  | 0.000 | 0.010 | 0.001 | 0.000 | 0.001 | 0.001 |  | 0.000 | 0.001 |  | 0.004 | 0.001 | |
| Gluconic acid | 0.603 | 0.866 | 0.421 | 2.054 | 0.876 | 0.410 |  | 0.480 | 5.405 | 1.049 | 0.798 | 1.405 | 0.818 |  | 0.630 | 1.113 |  | 2.311 | 1.007 | |
| 2-Oxoisovaleric acid | 0.936 | 0.873 | 0.358 | 4.828 | 0.718 | 0.470 |  | 0.405 | 4.007 | 0.987 | 1.140 | 1.477 | 0.930 |  | 0.722 | 2.005 |  | 1.800 | 1.182 | |
| cis-Aconitic acid | 0.013 | 0.002 | 0.006 | 0.033 | 0.002 | 0.003 |  | 0.002 | 0.002 | 0.003 | 0.006 | 0.002 | 0.005 |  | 0.007 | 0.013 |  | 0.002 | 0.004 | |
| Succinic acid | 0.306 | 0.134 | 0.118 | 0.604 | 0.115 | 0.103 |  | 0.088 | 0.415 | 0.184 | 0.134 | 0.178 | 0.244 |  | 0.186 | 0.274 |  | 0.229 | 0.185 | |
| Citric acid | 43.351 | 30.898 | 22.692 | 193.640 | 35.783 | 29.509 |  | 17.543 | 132.472 | 45.055 | 43.127 | 53.145 | 40.696 |  | 32.314 | 86.311 |  | 65.024 | 45.656 | |
| Shikimic acid | 0.001 | 0.001 | 0.000 | 0.005 | 0.001 | 0.001 |  | 0.001 | 0.007 | 0.001 | 0.002 | 0.000 | 0.001 |  | 0.001 | 0.002 |  | 0.003 | 0.001 | |
| Oxalic acid |  | 0.000 |  | 0.000 | 0.000 | 0.000 |  |  | 0.000 | 0.000 | 0.000 |  | 0.001 |  | 0.000 | 0.000 |  | 0.000 | 0.001 | |
| dCTP | 0.000 | 0.000 | 0.000 | 0.000 | 0.000 | 0.000 |  | 0.000 | 0.000 | 0.000 | 0.000 | 0.000 | 0.000 |  | 0.000 | 0.000 |  | 0.000 | 0.000 | |
| GDP | 0.000 | 0.001 | 0.000 | 0.000 | 0.000 | 0.000 |  | 0.001 | 0.001 | 0.001 | 0.000 | 0.000 | 0.001 |  | 0.000 | 0.000 |  | 0.001 | 0.000 | |
| GMP | 0.000 | 0.000 | 0.000 | 0.000 | 0.000 | 0.000 |  | 0.000 | 0.000 | 0.000 | 0.000 | 0.000 | 0.000 |  | 0.000 | 0.000 |  | 0.000 | 0.000 | |
| dATP | 0.001 | 0.002 | 0.002 | 0.001 | 0.001 | 0.001 |  | 0.002 | 0.001 | 0.001 | 0.001 | 0.001 | 0.001 |  | 0.002 | 0.001 |  | 0.001 | 0.001 | |
| CTP | 0.000 | 0.000 | 0.000 | 0.000 | 0.000 | 0.000 |  | 0.000 | 0.000 | 0.000 | 0.000 | 0.000 | 0.000 |  | 0.000 | 0.000 |  | 0.000 | 0.000 | |
| cGMP | 0.000 | 0.000 | 0.000 | 0.000 | 0.000 | 0.000 |  | 0.000 | 0.000 | 0.000 |  | 0.000 |  |  | 0.000 | 0.000 |  | 0.000 | 0.000 | |
| CMP | 0.006 | 0.009 | 0.011 | 0.009 | 0.007 | 0.007 |  | 0.010 | 0.008 | 0.006 | 0.009 | 0.009 | 0.008 |  | 0.009 | 0.008 |  | 0.008 | 0.008 | |
| UTP | 0.000 | 0.001 | 0.000 | 0.000 | 0.000 | 0.000 |  | 0.000 | 0.000 | 0.000 | 0.000 | 0.000 | 0.000 |  | 0.000 | 0.000 |  | 0.000 | 0.000 | |
| TTP | 0.000 | 0.001 | 0.000 | 0.000 | 0.000 | 0.000 |  | 0.000 | 0.000 | 0.000 | 0.000 | 0.000 | 0.001 |  | 0.000 | 0.000 |  | 0.000 | 0.000 | |
| UDP | 0.777 | 1.291 | 1.180 | 1.250 | 1.331 | 1.264 |  | 0.554 | 1.346 | 0.700 | 0.992 | 0.995 | 1.014 |  | 1.083 | 1.282 |  | 0.867 | 1.000 | |
| XMP | 0.000 | 0.000 | 0.000 | 0.000 | 0.000 | 0.000 |  | 0.000 | 0.000 | 0.000 | 0.000 | 0.000 | 0.000 |  | 0.000 | 0.000 |  | 0.000 | 0.000 | |
| UMP | 0.004 | 0.005 | 0.006 | 0.005 | 0.005 | 0.005 |  | 0.005 | 0.004 | 0.004 | 0.006 | 0.005 | 0.005 |  | 0.005 | 0.005 |  | 0.004 | 0.005 | |
| ATP | 0.010 | 0.016 | 0.018 | 0.012 | 0.015 | 0.017 |  | 0.009 | 0.017 | 0.009 | 0.013 | 0.014 | 0.019 |  | 0.014 | 0.015 |  | 0.012 | 0.015 | |
| ADP | 0.010 | 0.012 | 0.013 | 0.006 | 0.005 | 0.019 |  | 0.015 | 0.020 | 0.006 | 0.014 | 0.007 | 0.018 |  | 0.012 | 0.010 |  | 0.014 | 0.013 | |
| AMP | 0.002 | 0.001 | 0.004 | 0.005 | 0.003 | 0.002 |  | 0.003 | 0.004 | 0.002 | 0.004 | 0.004 | 0.003 |  | 0.003 | 0.003 |  | 0.003 | 0.004 | |
| GTP | 0.005 | 0.004 | 0.005 | 0.006 | 0.006 | 0.007 |  | 0.002 | 0.007 | 0.002 | 0.005 | 0.006 | 0.005 |  | 0.005 | 0.006 |  | 0.004 | 0.005 | |
| ITP | 0.000 | 0.001 | 0.001 | 0.000 | 0.001 | 0.001 |  | 0.001 | 0.000 | 0.000 | 0.000 | 0.000 | 0.001 |  | 0.000 | 0.001 |  | 0.001 | 0.000 | |
| TDP | 0.001 | 0.000 | 0.000 | 0.000 | 0.000 | 0.000 |  | 0.000 | 0.001 | 0.000 | 0.000 | 0.000 | 0.000 |  | 0.001 | 0.000 |  | 0.000 | 0.000 | |
| IMP | 0.000 | 0.000 | 0.000 | 0.000 | 0.000 | 0.000 |  | 0.000 | 0.000 | 0.000 | 0.001 | 0.001 | 0.000 |  | 0.000 | 0.000 |  | 0.000 | 0.001 | |
| NADH | 0.000 | 0.000 | 0.000 | 0.000 | 0.000 |  |  | 0.000 | 0.000 | 0.000 | 0.000 | 0.000 | 0.000 |  | 0.000 | 0.000 |  | 0.000 | 0.000 | |
| NAD+ | 0.002 | 0.002 | 0.005 | 0.001 | 0.001 | 0.002 |  | 0.003 | 0.003 | 0.001 | 0.002 | 0.003 | 0.003 |  | 0.003 | 0.001 |  | 0.002 | 0.003 | |
| Beta-Carotene | 0.199 | 0.324 | 4.592 | 0.273 | 0.306 | 3.293 |  | 0.250 | 0.318 | 7.705 | 0.271 | 0.182 | 6.456 |  | 1.705 | 1.291 |  | 2.757 | 2.303 | |
| Cryptoxanthin | 0.002 | 0.003 | 0.002 | 0.004 | 0.002 | 0.004 |  | 0.002 | 0.003 | 0.003 | 0.002 | 0.003 | 0.003 |  | 0.002 | 0.003 |  | 0.003 | 0.003 | |
| Xanthophyll | 16.093 | 26.524 | 14.280 | 24.860 | 17.549 | 13.959 |  | 19.383 | 23.797 | 24.899 | 30.800 | 23.662 | 22.454 |  | 18.966 | 18.789 |  | 22.693 | 25.639 | |
| Zeaxanthin | 6.479 | 10.534 | 5.731 | 10.019 | 7.209 | 5.612 |  | 6.634 | 9.770 | 10.106 | 11.965 | 9.870 | 8.871 |  | 7.581 | 7.614 |  | 8.837 | 10.235 | |
| Coenzyme Q10 | 0.000 | 0.001 | 0.000 | 0.000 | 0.000 | 0.001 |  | 0.000 | 0.000 | 0.000 | 0.001 | 0.001 | 0.001 |  | 0.001 | 0.000 |  | 0.000 | 0.001 | |
| chlorophyll a | 0.794 | 0.820 | 0.615 | 0.883 | 1.250 | 0.611 |  | 0.874 | 1.466 | 1.181 | 0.785 | 2.114 | 1.102 |  | 0.743 | 0.915 |  | 1.174 | 1.333 | |
| chlorophyll b | 0.636 | 1.063 | 0.467 | 0.791 | 0.818 | 0.464 |  | 0.780 | 1.248 | 0.976 | 0.938 | 1.273 | 0.742 |  | 0.722 | 0.691 |  | 1.001 | 0.984 | |
| Fructose | 0.055 | 0.070 | 0.039 | 0.082 | 0.087 | 0.032 |  | 0.081 | 0.077 | 0.056 | 0.077 | 0.101 | 0.054 |  | 0.054 | 0.067 |  | 0.071 | 0.077 | |
| Glucose | 0.165 | 0.168 | 0.085 | 0.209 | 0.192 | 0.060 |  | 0.185 | 0.173 | 0.128 | 0.210 | 0.257 | 0.138 |  | 0.139 | 0.153 |  | 0.162 | 0.202 | |
| Inositol | 0.333 | 0.358 | 0.281 | 0.302 | 0.308 | 0.303 |  | 0.301 | 0.358 | 0.343 | 0.281 | 0.305 | 0.371 |  | 0.324 | 0.305 |  | 0.334 | 0.319 | |
| Glucopyranose | 0.039 | 0.054 | 0.033 | 0.058 | 0.060 | 0.029 |  | 0.052 | 0.055 | 0.060 | 0.053 | 0.070 | 0.046 |  | 0.042 | 0.049 |  | 0.056 | 0.057 | |
| Glucopyranoside | 0.598 | 0.625 | 0.585 | 0.640 | 0.629 | 0.525 |  | 0.710 | 0.651 | 0.623 | 0.654 | 0.609 | 0.615 |  | 0.602 | 0.598 |  | 0.661 | 0.626 | |
| Xylose | 0.002 | 0.002 | 0.002 | 0.003 | 0.012 | 0.001 |  | 0.004 | 0.003 | 0.003 | 0.004 | 0.003 | 0.002 |  | 0.002 | 0.005 |  | 0.003 | 0.003 | |
| Myo-Inisitol | 0.005 | 0.006 | 0.003 | 0.008 | 0.008 | 0.003 |  | 0.007 | 0.007 | 0.008 | 0.008 | 0.010 | 0.005 |  | 0.005 | 0.006 |  | 0.007 | 0.008 | |
| Melibiose | 0.003 | 0.003 | 0.002 | 0.003 | 0.003 | 0.001 |  | 0.003 | 0.003 | 0.004 | 0.004 | 0.004 | 0.003 |  | 0.002 | 0.003 |  | 0.003 | 0.003 | |
| ***p*-value (diff sampling point)** | **0.926** | **0.581** | **0.679** | **0.316** | **0.741** | **0.274** |  | **0.276** | **0.435** | **0.543** | **0.985** | **0.824** | **0.802** |  |  |  |  |  |  | |
| ***p*-value (F12 vs F17)** | **0.324** | **0.920** | **0.912** |  |  |  |  | **0.438** | **0.490** | **0.930** |  |  |  |  | **0.493** |  |  | **0.815** |  | |

Quantified by relative abundance to the internal standard, Ribitol (2 mg mL^-1^).

T1, T2 and T3 are different sampling occasion with one month laps
