# Supplementary material for: Diurnal biomarkers reveal key photosynthetic genes associated with increased oil palm yield
Source: PLoS One. 2019 Mar 11;14(3):e0213591. doi: 10.1371/journal.pone.0213591 (PMC6411157; doi:10.1371/journal.pone.0213591)
Supplement: S3 Table — (DOCX) [file pone.0213591.s006.docx]

**Supplementary Table 3**

Metabolites concentration comparison of HY and LY palms

| Compound name | LY | | | | | HY | | | | |
| --- | --- | --- | --- | --- | --- | --- | --- | --- | --- | --- |
|  | 07:00 | 11:00 | 15:00 | 19:00 | 07:00 | 07:00 | 11:00 | 15:00 | 19:00 | 07:00 |
|  |  |  |  |  |  |  |  |  |  |  |
| **Glycolytic** |  |  |  |  |  |  |  |  |  |  |
| D-Fructose | 0.0823237 | 0.0615425 | 0.1110093 | 0.0669012 | 0.025267 | 0.074258 | 0.0812241 | 0.0931639 | 0.0335089 | 0.0166532 |
| d-Glucose | 0.1969914 | 0.1555486 | 0.1749249 | 0.1336957 | 0.0915132 | 0.1535093 | 0.1780923 | 0.1681738 | 0.0722993 | 0.0698648 |
| Glucose 1-phosphate | 6.177E-05 | 0.0001023 | 0.0001205 | 8.604E-05 | 6.432E-05 | 7.295E-05 | 0.0001054 | 0.0001387 | 9.13E-05 | 6.664E-05 |
| Glucose 6-phosphate | 0.0010612 | 0.0013988 | 0.0017498 | 0.0012757 | 0.0011858 | 0.0011971 | 0.0015373 | 0.0021216 | 0.0014106 | 0.0012197 |
| Fructose 6-phosphate | 0.000225 | 0.0003074 | 0.0003406 | 0.0002678 | 0.0002441 | 0.0002646 | 0.0002926 | 0.0004075 | 0.0002853 | 0.0002428 |
| Glycerol 3-phosphate | 0.0001326 | 0.0001705 | 0.0001757 | 0.0002248 | 0.0001545 | 0.0001314 | 0.0001666 | 0.0002159 | 0.0002363 | 0.0001553 |
| Phosphoenolpyruvic acid | 0.0002866 | 0.0003363 | 0.0005416 | 0.0001946 | 0.0002307 | 0.0003123 | 0.0004172 | 0.0005321 | 0.0002247 | 0.0002802 |
|  |  |  |  |  |  |  |  |  |  |  |
| **Pentose Phosphate** |  |  |  |  |  |  |  |  |  |  |
| Sucrose | 0.5228904 | 0.5182882 | 0.6604229 | 0.5945545 | 0.5490524 | 0.5242286 | 0.5823494 | 0.6739427 | 0.6178069 | 0.5620911 |
| Ribulose 1,5-diphosphate | 5.008E-05 | 0.0001479 | 0.0001231 | 0.0001404 |  | 5.717E-05 | 0.0001902 | 0.0001946 | 0.000141 | 1.905E-05 |
| Shikimic acid | 0.0001372 | 0.0001857 | 0.0001932 | 0.0001847 | 0.0001067 | 0.0001773 | 0.0001947 | 0.0002372 | 0.0001623 | 0.0001421 |
|  |  |  |  |  |  |  |  |  |  |  |
| **Sugar phosphates/ Sugar/ Sugar alcohol** |  |  |  |  |  |  |  |  |  |  |
| myo-Inositol 2-phosphate | 6.575E-05 | 6.896E-05 | 6.587E-05 | 6.604E-05 | 5.74E-05 | 7.004E-05 | 6.865E-05 | 6.952E-05 | 6.045E-05 | 5.554E-05 |
| myo-Inositol 1-phosphate myo-Inositol 3-phosphate | 0.0001656 | 0.0001907 | 0.0001483 | 0.0001541 | 0.0001106 | 0.000174 | 0.0001787 | 0.0001595 | 0.0001285 | 0.0001091 |
| Sedoheptulose 7-phosphate | 6.452E-05 | 0.0001359 | 0.0001619 | 9.38E-05 | 9.609E-05 | 7.454E-05 | 0.0001554 | 0.0001989 | 0.0001022 | 9.21E-05 |
| N-Acetylglucosamine 6-phosphate | 2.534E-05 | 2.707E-05 | 3.209E-05 | 2.104E-05 | 2.478E-05 | 2.813E-05 | 2.928E-05 | 3.546E-05 | 2.576E-05 | 2.81E-05 |
| N-Acetylglucosamine 1-phosphate | 2.577E-05 | 2.514E-05 | 2.063E-05 | 2.134E-05 | 1.872E-05 | 2.287E-05 | 2.374E-05 | 2.204E-05 | 1.818E-05 | 1.815E-05 |
| Sucrose 6'-phosphate | 0.0001023 | 0.0001028 | 0.0001221 | 0.0001221 | 0.0001068 | 0.0001176 | 0.0001187 | 0.0001298 | 0.0001238 | 0.0001315 |
| Trehalose 6-phosphate | 3.686E-05 | 6.497E-05 | 0.000121 | 9.764E-05 | 4.381E-05 | 3.485E-05 | 5.879E-05 | 0.0001164 | 0.000108 | 4.778E-05 |
| Glucopyranose phosphate | 0.001974 | 0.0005932 | 0.0034067 | 0.0015485 | 0.0004945 | 0.001054 | 0.0009221 | 0.0014233 | 0.0009289 | 0.0002278 |
| d-Glucopyranoside-ethyl | 0.031029 | 0.0190797 | 0.0263299 | 0.0617733 | 0.0141155 | 0.0200765 | 0.0144579 | 0.0374987 | 0.0186872 | 0.0044741 |
| D-Galactose | 0.0090166 | 0.0047806 | 0.0082914 | 0.0037896 | 0.0016991 | 0.0066061 | 0.0054794 | 0.0049522 | 0.0017258 | 0.0009365 |
| D-Xylose | 0.0032084 | 0.0023339 | 0.0025872 | 0.0011979 | 0.0007957 | 0.0022616 | 0.0018855 | 0.0017466 | 0.0012313 | 0.0005855 |
| Glycoside | 0.0034456 | 0.0016471 | 0.0030602 | 0.0008974 | 0.000706 | 0.0023662 | 0.0022446 | 0.001391 | 0.0013221 | 0.0005544 |
| Glucopyranose | 0.065274 | 0.029839 | 0.0554061 | 0.0527806 | 0.0171388 | 0.0428671 | 0.040727 | 0.0329508 | 0.0197906 | 0.0103973 |
| Inositol | 0.3533716 | 0.2880902 | 0.3178606 | 0.322741 | 0.2691747 | 0.3312266 | 0.3011245 | 0.3348157 | 0.2534974 | 0.2786209 |
|  |  |  |  |  |  |  |  |  |  |  |
| **TCA Cycle** |  |  |  |  |  |  |  |  |  |  |
| Malic acid | 0.0318747 | 0.0404115 | 0.0359593 | 0.0346541 | 0.0254636 | 0.0376237 | 0.0398134 | 0.0413451 | 0.0336111 | 0.0251495 |
| Citric acid | 0.0480803 | 0.0305161 | 0.0283524 | 0.0288797 | 0.0279488 | 0.0316716 | 0.0268117 | 0.0249128 | 0.0219144 | 0.0247926 |
| cis-Aconitic acid | 0.0003326 | 0.0002272 | 0.0002382 | 0.0002894 | 0.000264 | 0.0003075 | 0.0002364 | 0.0002705 | 0.0002661 | 0.0002474 |
| Isocitric acid | 0.0029881 | 0.0027908 | 0.0025434 | 0.0024468 | 0.0021678 | 0.0025493 | 0.0022008 | 0.0020785 | 0.0017373 | 0.0017397 |
| Succinic acid | 0.002125 | 0.0031804 | 0.0031235 | 0.0024007 | 0.0018236 | 0.0026032 | 0.0036432 | 0.0033212 | 0.0025139 | 0.0024289 |
| Fumaric acid | 7.089E-05 | 0.0001927 | 8.721E-05 | 0.0001158 | 7.271E-05 | 6.261E-05 | 0.0001059 | 0.0001017 | 9.512E-05 | 9.143E-05 |
|  |  |  |  |  |  |  |  |  |  |  |
| **Amino acid** |  |  |  |  |  |  |  |  |  |  |
| Ser | 0.0284801 | 0.0218375 | 0.0236567 | 0.025859 | 0.0318715 | 0.0364317 | 0.0385861 | 0.0329469 | 0.0338532 | 0.0336867 |
| Gly | 0.0003426 | 0.0006627 | 0.0007537 | 0.0003537 | 0.0003696 | 0.0003576 | 0.0008492 | 0.0007975 | 0.000391 | 0.0003793 |
| β-Ala | 0.0001827 | 0.0001197 | 0.0001083 | 0.0001166 | 0.0002449 | 0.0001955 | 0.0001496 | 0.0001384 | 0.0001255 | 0.0002606 |
| Ala | 0.0211903 | 0.0346748 | 0.0302145 | 0.0223635 | 0.0237171 | 0.020838 | 0.0340776 | 0.0321907 | 0.0239536 | 0.0220106 |
| Leu | 0.0013064 | 0.0013844 | 0.0010715 | 0.002439 | 0.0023452 | 0.0010514 | 0.0018182 | 0.0013119 | 0.0020893 | 0.0019224 |
| Ile | 0.0018538 | 0.0021655 | 0.0019577 | 0.0025492 | 0.0032083 | 0.0015544 | 0.0026223 | 0.0024761 | 0.0024166 | 0.0026701 |
| Val | 0.0037256 | 0.0043954 | 0.004405 | 0.0042758 | 0.0057642 | 0.0026285 | 0.0046335 | 0.0041607 | 0.0040232 | 0.0045388 |
| Glu | 0.0769269 | 0.0837174 | 0.0910232 | 0.0762584 | 0.0747662 | 0.0756102 | 0.0884833 | 0.084242 | 0.0778356 | 0.0681147 |
| Gln | 0.0063008 | 0.00848 | 0.0100861 | 0.0074142 | 0.0089337 | 0.0045827 | 0.0074498 | 0.0106546 | 0.0086537 | 0.0073633 |
| Pro | 0.0108221 | 0.0103382 | 0.0103796 | 0.0104912 | 0.011984 | 0.0098897 | 0.0108168 | 0.009857 | 0.0103173 | 0.0116423 |
| Arg | 0.0046526 | 0.0037033 | 0.0018314 | 0.0027257 | 0.0031418 | 0.0036806 | 0.0044107 | 0.0022495 | 0.00218 | 0.0029098 |
| Asp | 0.0526188 | 0.0397352 | 0.0507158 | 0.0522475 | 0.048726 | 0.0544421 | 0.0465836 | 0.044344 | 0.0537051 | 0.0526063 |
| Asn | 0.0030243 | 0.0029042 | 0.0019439 | 0.0019643 | 0.0021769 | 0.0048152 | 0.0054271 | 0.0029853 | 0.0021938 | 0.0022953 |
| Thr | 0.0049624 | 0.0051363 | 0.0060998 | 0.0059287 | 0.006338 | 0.005268 | 0.0069409 | 0.0072439 | 0.0075662 | 0.0070794 |
| Met | 7.635E-05 | 0.0001871 | 0.000132 | 0.0001324 | 0.0001282 | 5.511E-05 | 0.0001789 | 0.0001155 | 0.0001249 | 9.836E-05 |
| Ornithine | 6.372E-05 | 6.098E-05 | 5.736E-05 | 8.445E-05 | 6.037E-05 | 4.046E-05 | 5.845E-05 | 6.212E-05 | 7.229E-05 | 4.036E-05 |
| Cysteic acid | 3.535E-05 | 3.215E-05 | 3.97E-05 | 3.832E-05 | 4.016E-05 | 3.641E-05 | 3.536E-05 | 3.828E-05 | 4.024E-05 | 3.994E-05 |
| His | 0.0009051 | 0.0006599 | 0.0006725 | 0.000848 | 0.0011373 | 0.0006636 | 0.0008114 | 0.0006623 | 0.0007816 | 0.0010862 |
| Phe | 0.000918 | 0.0009698 | 0.0008923 | 0.0015815 | 0.0019972 | 0.0006078 | 0.0011303 | 0.0011066 | 0.0013483 | 0.0018243 |
| Tyr | 0.0006079 | 0.0005451 | 0.0004079 | 0.0007046 | 0.0008799 | 0.0004529 | 0.0006446 | 0.0004285 | 0.0005371 | 0.0007604 |
| Tyrosine methyl ester | 0.0001603 | 0.0001072 | 0.0001333 | 0.0001176 | 0.0001299 | 0.0001401 | 0.0001598 | 0.0001102 | 0.0001036 | 9.491E-05 |
| Tyramine | 0.0675945 | 0.071096 | 0.0523425 | 0.0465927 | 0.049262 | 0.0580266 | 0.0657851 | 0.041847 | 0.0389756 | 0.0257174 |
| Trp | 0.0009393 | 0.0006121 | 0.0010434 | 0.0013693 | 0.0013154 | 0.0007429 | 0.0009113 | 0.0007937 | 0.0010691 | 0.0010639 |
| Lys | 0.0010136 | 0.0010152 | 0.0006237 | 0.0008148 | 0.0019752 | 0.0007598 | 0.0011298 | 0.0005936 | 0.0007516 | 0.0014858 |
| Glutathione (GSSG)_divalent | 8.765E-05 | 8.676E-05 | 0.0001284 | 9.086E-05 | 9.539E-05 | 7.359E-05 | 0.0001209 | 0.0001327 | 8.851E-05 | 9.243E-05 |
| DOPA | 2.976E-05 | 4.275E-05 | 6.211E-05 | 6.376E-05 | 7.809E-05 | 2.501E-05 | 6.33E-05 | 6.008E-05 | 4.533E-05 | 6.615E-05 |
| Hydroxyproline | 0.0002679 | 0.0002671 | 0.0003202 | 0.0002786 | 0.0002741 | 0.0002108 | 0.0002545 | 0.0002776 | 0.0002842 | 0.00027 |
|  |  |  |  |  |  |  |  |  |  |  |
| **Polyamines** |  |  |  |  |  |  |  |  |  |  |
| Putrescine | 0.0004393 | 0.0004501 | 0.0005125 | 0.0005737 | 0.0006879 | 0.0005316 | 0.0004413 | 0.0005121 | 0.0005397 | 0.0006152 |
| Citrulline | 7.344E-05 | 8.522E-05 | 7.706E-05 | 6.031E-05 | 4.31E-05 | 0.0001232 | 0.0001436 | 0.0001959 | 0.0001513 | 8.947E-05 |
| S-Adenosylmethionine | 6.155E-05 | 3.801E-05 | 4.915E-05 | 7.025E-05 | 0.0001104 | 5.89E-05 | 5.887E-05 | 5.695E-05 | 6.963E-05 | 0.0001056 |
| Spermidine | 0.0008844 | 0.000882 | 0.0009366 | 0.0008569 | 0.0008547 | 0.0008617 | 0.0009743 | 0.0008616 | 0.0009925 | 0.0007817 |
| Spermine | 4.111E-05 | 3.616E-05 | 3.953E-05 | 3.243E-05 | 3.02E-05 | 4.777E-05 | 5.035E-05 | 3.615E-05 | 4.167E-05 | 3.205E-05 |
| GABA | 0.0081418 | 0.0109596 | 0.0087764 | 0.0107363 | 0.0091007 | 0.0074912 | 0.0103513 | 0.0113106 | 0.0115422 | 0.0066787 |
|  |  |  |  |  |  |  |  |  |  |  |
|  |  |  |  |  |  |  |  |  |  |  |
| **Glycolipids synthesis** |  |  |  |  |  |  |  |  |  |  |
| 3-Aminopropane-1,2-diol | 7.581E-05 | 7.291E-05 | 8.578E-05 | 8.708E-05 | 8.573E-05 | 7.765E-05 | 8.282E-05 | 8.385E-05 | 9.213E-05 | 9.512E-05 |
| Phosphorylcholine | 0.0002831 | 0.0003493 | 0.0002639 | 0.0002073 | 0.0003527 | 0.0003084 | 0.0003784 | 0.0003161 | 0.0002041 | 0.0004561 |
| Choline | 0.0474857 | 0.0462398 | 0.0487388 | 0.0454833 | 0.0445886 | 0.0422724 | 0.0440917 | 0.0460257 | 0.0440147 | 0.0432795 |
| Glycerol | 0.002772 | 0.0024018 | 0.0027817 | 0.0022014 | 0.0029052 | 0.002647 | 0.003297 | 0.0023004 | 0.0024513 | 0.0022863 |
| Glycerophosphocholine | 0.0005535 | 0.0005675 | 0.0005972 | 0.0005733 | 0.0005176 | 0.000529 | 0.0005223 | 0.0006589 | 0.0005461 | 0.0006365 |
|  |  |  |  |  |  |  |  |  |  |  |
| **Ascorbic acid metabolism** |  |  |  |  |  |  |  |  |  |  |
| Glucaric acid | 0.0076634 | 0.0077365 | 0.0088476 | 0.0080889 | 0.0087718 | 0.0074771 | 0.0079888 | 0.008063 | 0.0078935 | 0.0078119 |
| Gluconolactone | 0.0001287 | 0.0001474 | 0.0001192 | 0.0001172 | 0.0001094 | 0.0001321 | 0.0001324 | 0.000129 | 0.0001115 | 9.537E-05 |
| Gulonolactone | 0.0002171 | 0.0002076 | 0.0002176 | 0.0002254 | 0.0002176 | 0.0001926 | 0.0001906 | 0.0001989 | 0.0001935 | 0.0001924 |
| Tartaric acid | 0.000122 | 0.0001221 | 9.677E-05 | 8.15E-05 | 9.634E-05 | 0.0001547 | 0.0001165 | 0.0001205 | 7.709E-05 | 9.044E-05 |
| Gluconic acid | 0.001031 | 0.0010898 | 0.0010269 | 0.0009783 | 0.0008632 | 0.0010015 | 0.0009699 | 0.0009777 | 0.0008449 | 0.0008372 |
| Ascorbate 2-glucoside | 0.0006891 | 0.0006885 | 0.0007125 | 0.0008361 | 0.0008129 | 0.0009858 | 0.0010688 | 0.0009901 | 0.0010919 | 0.001088 |
| Threonic acid | 0.0038872 | 0.0032223 | 0.0045988 | 0.0042113 | 0.0040138 | 0.0035435 | 0.0034291 | 0.0039316 | 0.0039857 | 0.0035516 |
| Ethyl glucuronide | 8.558E-05 | 8.986E-05 | 9.367E-05 | 8.16E-05 | 9.027E-05 | 9.353E-05 | 7.506E-05 | 0.0001025 | 8.917E-05 | 9.435E-05 |
| Glucurone | 4.629E-05 | 9.551E-05 | 6.857E-05 | 0.0001068 | 0.0001034 | 0.0001366 | 0.0001329 | 0.0001374 | 0.0001957 | 9.042E-05 |
|  |  |  |  |  |  |  |  |  |  |  |
| **Organic acid** |  |  |  |  |  |  |  |  |  |  |
| Crotonic acid | 0.0008399 | 0.0008184 | 0.001086 | 0.0010304 | 0.0010608 | 0.0008139 | 0.0009091 | 0.0009294 | 0.0009863 | 0.000969 |
| Lactic acid | 0.000302 | 0.0003052 | 0.0003604 | 0.0003352 | 0.0019711 | 0.0003116 | 0.0004495 | 0.0004238 | 0.000385 | 0.0002529 |
| Glyceric acid | 0.0003858 | 0.000828 | 0.001383 | 0.000494 | 0.000323 | 0.0003181 | 0.0009374 | 0.0014692 | 0.0005022 | 0.0003022 |
|  |  |  |  |  |  |  |  |  |  |  |
| **Nucleosides/ Nucleobases** |  |  |  |  |  |  |  |  |  |  |
| Adenine | 0.0007818 | 0.0009879 | 0.0007603 | 0.0006163 | 0.0005149 | 0.0006709 | 0.0005426 | 0.0007638 | 0.0003231 | 0.0005697 |
| Adenosine | 0.0002632 | 0.0003607 | 0.0004279 | 0.0001916 | 0.0002525 | 0.000315 | 0.0003777 | 0.0003419 | 0.0001666 | 0.0002342 |
| Cytidine | 3.806E-05 | 4.853E-05 | 4.609E-05 | 3.279E-05 | 2.94E-05 | 3.247E-05 | 3.426E-05 | 4.118E-05 | 2.929E-05 | 3.216E-05 |
| Cytosine | 7.111E-05 | 6.508E-05 | 8.077E-05 | 7.481E-05 | 7.708E-05 | 7.184E-05 | 7.38E-05 | 8.446E-05 | 8.68E-05 | 9.295E-05 |
| Guanosine | 8.979E-05 | 0.0001268 | 9.901E-05 | 8.531E-05 | 7.242E-05 | 8.82E-05 | 8.074E-05 | 9.93E-05 | 5.93E-05 | 7.069E-05 |
| Thiamine | 2.457E-05 | 1.672E-05 | 1.753E-05 | 1.905E-05 | 2.487E-05 | 2.136E-05 | 2.076E-05 | 1.781E-05 | 2.13E-05 | 1.993E-05 |
|  |  |  |  |  |  |  |  |  |  |  |
| **Nucleotides** |  |  |  |  |  |  |  |  |  |  |
| ADP | 0.0002791 | 0.000289 | 0.0003609 | 0.0002447 | 0.0002607 | 0.0003289 | 0.0004283 | 0.0004028 | 0.000297 | 0.0002924 |
| ATP | 0.000167 | 0.0002344 | 0.0002088 | 0.0002615 | 0.00017 | 0.0002227 | 0.0003122 | 0.0002817 | 0.0002914 | 0.0002049 |
| GDP | 2.324E-05 | 2.459E-05 | 3.001E-05 | 2.08E-05 | 1.988E-05 | 2.776E-05 | 3.527E-05 | 3.141E-05 | 2.07E-05 | 2.328E-05 |
| NAD+ | 0.000121 | 9.407E-05 | 0.000123 | 0.0001106 | 0.0001487 | 0.0001267 | 0.0001159 | 0.0001235 | 0.0001321 | 0.0001452 |
| NADP+ | 5.28E-05 | 5.733E-05 | 7.241E-05 | 5.825E-05 | 4.434E-05 | 5.926E-05 | 8.253E-05 | 7.344E-05 | 7.197E-05 | 4.709E-05 |
| UDP | 5.895E-05 | 5.268E-05 | 6.537E-05 | 4.758E-05 | 4.948E-05 | 5.829E-05 | 7.999E-05 | 7.389E-05 | 5.913E-05 | 5.397E-05 |
| UDP-glucose UDP-galactose | 0.0006464 | 0.0007005 | 0.0008037 | 0.0007411 | 0.0006166 | 0.0007176 | 0.0007971 | 0.0008577 | 0.0007947 | 0.0006761 |
| UDP-N-acetylglucosamine | 7.553E-05 | 7.692E-05 | 6.945E-05 | 7.246E-05 | 6.66E-05 | 7.017E-05 | 7.416E-05 | 7.045E-05 | 6.374E-05 | 7.319E-05 |
| UTP | 6.626E-05 | 7.345E-05 | 6.704E-05 | 0.0001028 | 6.294E-05 | 7.136E-05 | 8.928E-05 | 7.855E-05 | 0.0001048 | 6.347E-05 |
|  |  |  |  |  |  |  |  |  |  |  |
| **Hormone** |  |  |  |  |  |  |  |  |  |  |
| Adrenaline | 0.0004845 | 0.0003098 | 0.0001774 | 0.0001651 | 0.000127 | 0.00031 | 0.000223 | 0.0001865 | 7.747E-05 | 7.631E-05 |
| Serotonin | 0.0001072 | 0.0002166 | 0.0004425 | 0.0004972 | 0.0007351 | 8.446E-05 | 0.0004145 | 0.0015428 | 0.0015827 | 0.0016544 |
|  |  |  |  |  |  |  |  |  |  |  |
| **Others** |  |  |  |  |  |  |  |  |  |  |
| 2-Aminoadipic acid | 8.201E-05 | 6.406E-05 | 0.0001077 | 0.0001064 | 0.0001232 | 6.429E-05 | 0.0001254 | 0.000118 | 0.0001228 | 0.0001412 |
| 2-Aminobutyric acid | 0.0001225 | 0.0001142 | 0.0001354 | 0.00015 | 0.000104 | 0.0001093 | 0.000122 | 0.0001396 | 0.0001326 | 9.876E-05 |
| 2-Methylserine | 0.0001392 | 0.0001366 | 0.0001356 | 0.0001364 | 0.0001212 | 0.0001616 | 0.0001594 | 0.0001536 | 0.0001283 | 0.000133 |
| 355.09952/9.068_N | 0.1207641 | 0.1290653 | 0.1386253 | 0.1490021 | 0.1237625 | 0.1723649 | 0.1899818 | 0.2140815 | 0.2186331 | 0.2281073 |
| 3-Methyladenine | 0.0001119 | 9.895E-05 | 0.0001335 | 0.0001245 | 0.0001159 | 0.0001281 | 0.0001188 | 0.0001211 | 0.0001141 | 0.0001219 |
| 458.18784/1.34_P | 0.0030505 | 0.0058279 | 0.0057335 | 0.0052852 | 0.0050099 | 0.0030215 | 0.0092862 | 0.0062267 | 0.007037 | 0.0053044 |
| 4-Guanidinobutyric acid | 0.0001379 | 0.0001335 | 0.0001573 | 0.0001453 | 0.0001634 | 0.0001506 | 0.0001504 | 0.000178 | 0.0001816 | 0.0001889 |
| 5-Aminovaleric acid | 7.909E-05 | 0.0001024 | 0.0001054 | 8.506E-05 | 8.377E-05 | 9.053E-05 | 0.000103 | 0.0001009 | 9.512E-05 | 8.454E-05 |
| 5-Methylcytosine | 2.835E-05 | 2.664E-05 | 3.12E-05 | 2.994E-05 | 2.917E-05 | 3.019E-05 | 3.038E-05 | 3.134E-05 | 3.139E-05 | 3.3E-05 |
| 5-Oxo-2-tetrahydrofurancarboxylic acid | 0.0002345 | 0.0002274 | 0.0002999 | 0.0002887 | 0.0003219 | 0.0002252 | 0.000254 | 0.0002516 | 0.0002768 | 0.0002811 |
| 5-Oxoproline | 8.624E-05 | 8.509E-05 | 8.958E-05 | 8.27E-05 | 0.0001568 | 8.049E-05 | 0.0001165 | 9.961E-05 | 9.628E-05 | 7.779E-05 |
| 6,8-Thioctic acid | 0.0005886 | 0.0006336 | 0.0005777 | 0.0006151 | 0.0005398 | 0.0007325 | 0.0007179 | 0.0006818 | 0.0005893 | 0.0007027 |
| ADMA | 2.943E-05 | 2.906E-05 | 2.684E-05 | 2.448E-05 | 2.697E-05 | 3.156E-05 | 3.494E-05 | 2.721E-05 | 2.214E-05 | 2.223E-05 |
| Ascorbate 2-sulfate | 0.0001481 | 0.0001499 | 0.0001358 | 0.0001338 | 0.0001308 | 0.0001437 | 0.0001301 | 0.0001288 | 0.0001213 | 0.0001213 |
| Betaine | 0.0037578 | 0.0041252 | 0.0035473 | 0.00414 | 0.0035931 | 0.0028144 | 0.0032216 | 0.003341 | 0.0030614 | 0.003565 |
| Butyrylcarnitine | 2.798E-05 | 4.135E-05 | 3.973E-05 | 3.317E-05 | 2.591E-05 | 2.028E-05 | 2.951E-05 | 3.957E-05 | 3.405E-05 | 2.781E-05 |
| Carnitine | 0.0001933 | 0.0002527 | 0.0002095 | 0.0002296 | 0.0002108 | 0.0001233 | 0.0001805 | 0.0001807 | 0.0001729 | 0.0002125 |
| Chelidonic acid | 0.2066299 | 0.2012232 | 0.2004835 | 0.1983086 | 0.1937037 | 0.2114057 | 0.2136611 | 0.1965365 | 0.1933587 | 0.1798064 |
| cis-4-Hydroxyproline | 6.977E-05 | 7.126E-05 | 7.733E-05 | 7.701E-05 | 7.769E-05 | 8.16E-05 | 8.291E-05 | 7.896E-05 | 7.509E-05 | 7.046E-05 |
| Citramalic acid | 0.0001276 | 0.0001276 | 0.0001372 | 0.0001148 | 8.901E-05 | 0.0001129 | 0.0001216 | 0.0001527 | 0.0001129 | 9.746E-05 |
| Cyclohexylamine | 2.468E-05 | 1.618E-05 | 1.691E-05 | 1.79E-05 | 1.706E-05 | 1.726E-05 | 1.774E-05 | 1.758E-05 | 1.782E-05 | 1.528E-05 |
| Diethanolamine | 3.759E-05 | 3.007E-05 | 3.287E-05 | 3.606E-05 | 3.609E-05 | 2.831E-05 | 3.274E-05 | 3.348E-05 | 3.82E-05 | 3.334E-05 |
| Ethanolamine | 0.0112886 | 0.010256 | 0.0111098 | 0.010685 | 0.0105718 | 0.010138 | 0.0101544 | 0.0103713 | 0.0104984 | 0.0108812 |
| Ethanolamine phosphate | 5.855E-05 | 9.638E-05 | 6.397E-05 | 3.952E-05 | 8.029E-05 | 8.169E-05 | 0.0001134 | 7.774E-05 | 3.402E-05 | 8.692E-05 |
| Hexylamine | 3.894E-05 | 4.421E-05 | 3.597E-05 | 3.79E-05 | 4.144E-05 | 3.576E-05 | 3.634E-05 | 3.369E-05 | 3.542E-05 | 2.982E-05 |
| Histamine | 2.828E-05 | 2.561E-05 | 2.035E-05 | 1.804E-05 | 1.676E-05 | 2.018E-05 | 2.433E-05 | 1.711E-05 | 1.527E-05 | 1.264E-05 |
| Trimethylamine | 9.814E-05 | 0.0001159 | 0.0001092 | 0.0001025 | 0.0001238 | 0.0001115 | 0.0001165 | 0.000107 | 0.0001044 | 0.000109 |
| Imidazole-4-acetic acid | 3.039E-05 | 2.551E-05 | 3.518E-05 | 3.571E-05 | 3.333E-05 | 3.672E-05 | 3.399E-05 | 3.717E-05 | 4.067E-05 | 3.673E-05 |
| Imidazolelactic acid | 2.398E-05 | 2.521E-05 | 2.654E-05 | 2.679E-05 | 2.825E-05 | 2.487E-05 | 2.436E-05 | 2.716E-05 | 2.64E-05 | 2.845E-05 |
| Isethionic acid | 3.059E-05 | 2.854E-05 | 2.339E-05 | 2.369E-05 | 2.45E-05 | 2.897E-05 | 3.118E-05 | 2.566E-05 | 2.28E-05 | 2.069E-05 |
| Isobutylamine | 0.0002288 | 0.0001287 | 0.0001847 | 0.0001597 | 0.0001396 | 0.0002015 | 0.0001807 | 0.000199 | 0.0002347 | 0.0001546 |
| Kynurenic acid | 7.719E-05 | 6.863E-05 | 0.0001054 | 0.0001052 | 0.0001073 | 5.476E-05 | 7.149E-05 | 8.053E-05 | 8.321E-05 | 0.0001014 |
| Methyl sulfate | 0.0003653 | 0.0003831 | 0.0003546 | 0.0003263 | 0.0003271 | 0.0003027 | 0.0002662 | 0.0002644 | 0.0002571 | 0.000223 |
| Morpholine | 2.436E-05 | 2.325E-05 | 2.406E-05 | 2.928E-05 | 2.492E-05 | 5.488E-05 | 5.881E-05 | 4.734E-05 | 5.308E-05 | 5.236E-05 |
| Mucic acid | 0.0003007 | 0.00026 | 0.0003216 | 0.0002746 | 0.0003092 | 0.000248 | 0.0002659 | 0.0002707 | 0.0002574 | 0.0002402 |
| N5-Ethylglutamine | 0.0001353 | 0.0001285 | 0.0001451 | 0.0001406 | 0.0001467 | 8.284E-05 | 9.925E-05 | 0.0001093 | 0.0001157 | 0.0001028 |
| N6,N6,N6-Trimethyllysine | 8.326E-05 | 8.139E-05 | 7.082E-05 | 6.845E-05 | 6.452E-05 | 8.887E-05 | 9.73E-05 | 8.277E-05 | 7.529E-05 | 6.598E-05 |
| N-Acetylglutamic acid | 0.0002318 | 0.0001566 | 0.0001284 | 0.0002318 | 0.0001895 | 0.0002973 | 0.0001826 | 0.0001594 | 0.0001946 | 0.0002228 |
| N-Acetyllysine | 2.566E-05 | 2.784E-05 | 2.797E-05 | 2.617E-05 | 2.863E-05 | 2.272E-05 | 2.84E-05 | 2.755E-05 | 2.787E-05 | 3.071E-05 |
| N-Acetylputrescine | 0.0001994 | 0.0002177 | 0.0001786 | 0.0002002 | 0.0001815 | 0.0002564 | 0.0002461 | 0.0002055 | 0.0001784 | 0.0001809 |
| N-Methylalanine | 2.75E-05 | 2.771E-05 | 2.864E-05 | 3.05E-05 | 2.998E-05 | 2.204E-05 | 2.753E-05 | 2.701E-05 | 3.208E-05 | 3.263E-05 |
| N-Methylnorsalsolinol | 6.857E-05 | 6.733E-05 | 7.368E-05 | 7.345E-05 | 7.878E-05 | 8.226E-05 | 8.775E-05 | 7.796E-05 | 8.389E-05 | 8.297E-05 |
| Noradrenaline | 8.388E-05 | 8.647E-05 | 6.279E-05 | 5.554E-05 | 4.546E-05 | 7.028E-05 | 5.293E-05 | 5.592E-05 | 2.984E-05 | 2.938E-05 |
| Nω-Methylarginine | 0.0002395 | 0.0001629 | 6.626E-05 | 9.059E-05 | 6.484E-05 | 0.0001642 | 0.0001832 | 8.74E-05 | 5.67E-05 | 5.155E-05 |
| O-Acetylcarnitine | 0.0001304 | 0.0001429 | 0.0001598 | 0.0001614 | 0.0001531 | 8.614E-05 | 0.0001144 | 0.0001453 | 0.0001625 | 0.0001518 |
| O-Acetylserine | 8.98E-05 | 0.0001367 | 0.0001376 | 6.502E-05 | 7.023E-05 | 0.0001291 | 0.0001847 | 0.0001433 | 8.087E-05 | 0.0002 |
| Octopamine | 0.0180047 | 0.0130859 | 0.0125266 | 0.0114823 | 0.0100519 | 0.0149681 | 0.0215389 | 0.0091731 | 0.0099373 | 0.0067196 |
| Ophthalmic acid | #DIV/0! | 3.454E-05 | 4.561E-05 | 3.094E-05 | #DIV/0! | #DIV/0! | 3.061E-05 | 3.937E-05 | 2.523E-05 | #DIV/0! |
| Pantothenic acid | 0.000114 | 0.0001211 | 0.0001243 | 0.0001157 | 0.0001231 | 0.0001222 | 0.0001236 | 0.000141 | 0.0001396 | 0.0001318 |
| Pelargonic acid | 8.42E-05 | 9.235E-05 | 9.33E-05 | 9.275E-05 | 9.377E-05 | 8.718E-05 | 8.708E-05 | 7.414E-05 | 9.173E-05 | 8.137E-05 |
| Penciclovir | 0.0001154 | 0.0001021 | 0.0001405 | 0.0001466 | 0.0001395 | 0.0001101 | 0.0001102 | 0.0001225 | 0.0001319 | 0.0001491 |
| Pipecolic acid | 0.0077506 | 0.0057221 | 0.0068709 | 0.0079791 | 0.008781 | 0.0048221 | 0.0058345 | 0.0061245 | 0.0050571 | 0.006402 |
| Saccharopine | 4.89E-05 | 5.561E-05 | 6.345E-05 | 6.686E-05 | 7.725E-05 | 4.477E-05 | 6.717E-05 | 6.487E-05 | 6.57E-05 | 9.086E-05 |
| SDMA | 2.377E-05 | 2.381E-05 | 2.216E-05 | 1.833E-05 | 2.031E-05 | 2.511E-05 | 2.303E-05 | 2.1E-05 | 1.697E-05 | 1.543E-05 |
| Stachydrine | 0.0001852 | 0.0002431 | 0.0001385 | 0.0002046 | 9.445E-05 | 6.976E-05 | 0.0001218 | 7.738E-05 | 6.694E-05 | 8.289E-05 |
| Sulfotyrosine | 3.125E-05 | 2.806E-05 | 2.958E-05 | 2.957E-05 | 2.522E-05 | 3.003E-05 | 2.729E-05 | 2.715E-05 | 2.677E-05 | 2.622E-05 |
| Terephthalic acid | 9.57E-05 | 0.0001026 | 0.0001011 | 0.0001053 | 0.0001138 | 0.0001023 | 0.0001112 | 0.0001041 | 0.0001108 | 9.461E-05 |
| Theobromine | 0.0003553 | 0.0003635 | 0.0003213 | 0.0003217 | 0.0003669 | 0.0003459 | 0.000352 | 0.000304 | 0.0003184 | 0.0003125 |
| Trigonelline | 0.0408323 | 0.0417784 | 0.0417462 | 0.038027 | 0.0379611 | 0.0363127 | 0.0335278 | 0.0310113 | 0.0296136 | 0.0279363 |
| Trimethylamine N-oxide | 3.512E-05 | 4.014E-05 | 3.722E-05 | 4.269E-05 | 3.401E-05 | 3.799E-05 | 3.994E-05 | 4.036E-05 | 3.894E-05 | 3.521E-05 |
| Tryptamine | 0.0001622 | 0.0001534 | 0.0005494 | 0.0004485 | 0.000794 | 0.0001075 | 0.0002134 | 0.0003455 | 0.0005565 | 0.0006167 |

Quantified by relative abundance to the internal standard, Ribitol (2 mg mL^-1^)
