# Supplementary material for: Diurnal biomarkers reveal key photosynthetic genes associated with increased oil palm yield
Source: PLoS One. 2019 Mar 11;14(3):e0213591. doi: 10.1371/journal.pone.0213591 (PMC6411157; doi:10.1371/journal.pone.0213591)
Supplement: S4 Table — (DOCX) [file pone.0213591.s007.docx]

**Supplementary Table 4**

Total starch content comparison of HY and LY palms

|  | **LY** | **SD (LY)** | **HY** | **SD (HY)** | **p-value** |
| --- | --- | --- | --- | --- | --- |
| **07:00** | 0.0673667 | 0.0087778 | 0.07197 | 0.0304409 | 0.8136976 |
| **11:00** | 0.07012 | 0.018622 | 0.0866667 | 0.018201 | 0.3328427 |
| **15:00** | 0.08742 | 0.029115 | 0.0833633 | 0.022507 | 0.8578776 |
| **19:00** | 0.1076967 | 0.0238723 | 0.0790133 | 0.0101912 | 0.1281544 |
| **07:00** | 0.05146 | 0.0176243 | 0.0567933 | 0.0239584 | 0.7716266 |

**Method reference**

Chow, P.S. and Landhäusser, S.M. (2004) A method for routine measurements of total sugar and starch content in woody plant tissues. *Tree Physiol.* *24*, 1129 –1136
