# Supplementary material for: Diurnal biomarkers reveal key photosynthetic genes associated with increased oil palm yield
Source: PLoS One. 2019 Mar 11;14(3):e0213591. doi: 10.1371/journal.pone.0213591 (PMC6411157; doi:10.1371/journal.pone.0213591)
Supplement: S5 Table — (DOCX) [file pone.0213591.s008.docx]

**Supplementary Table 5**

Differentially expressed genes list for individual diurnal time point from transcriptome sequencing

07:00

| **GeneID** | **log_2_Ratio** | **Q value** | **Annotation** |
| --- | --- | --- | --- |
| isotig65188 | 11.39713 | 0.970884 | sp\|Q9LRR5\|DRL21_ARATH Putative disease resistance protein At3g14460 OS=Arabidopsis thaliana GN=At3g14460 PE=2 SV=1 length=367 |
| isotig42424 | 9.94241 | 0.985419 | NO_ANNOT_AVAILABLE length=621 |
| isotig35527 | 9.81319 | 0.91516 | sp\|Q5RBU7\|PCP_PONAB Lysosomal Pro-X carboxypeptidase OS=Pongo abelii GN=PRCP PE=2 SV=1 length=827 |
| isotig22997 | 8.92968 | 0.860171 | NO_ANNOT_AVAILABLE length=597 |
| isotig59762 | 8.78090 | 0.848876 | NO_ANNOT_AVAILABLE length=496 |
| isotig67143 | 8.66108 | 0.839558 | sp\|P82413\|RK19_SPIOL 50S ribosomal protein L19, chloroplastic OS=Spinacia oleracea GN=RPL19 PE=1 SV=2 length=331 |
| isotig70620 | 8.49808 | 0.826363 | NO_ANNOT_AVAILABLE length=262 |
| isotig72193 | 8.44227 | 0.821649 | NO_ANNOT_AVAILABLE length=229 |
| isotig56650 | 7.59489 | 0.899497 | NO_ANNOT_AVAILABLE length=612 |
| isotig21342 | 6.66256 | 0.852713 | NO_ANNOT_AVAILABLE length=975 |
| isotig21040 | 6.58803 | 0.82407 | sp\|P27489\|CB23_SOLLC Chlorophyll a-b binding protein 13, chloroplastic OS=Solanum lycopersicum GN=CAB13 PE=1 SV=1 length=1116 |
| isotig69058 | 5.78070 | 0.944788 | NO_ANNOT_AVAILABLE length=287 |
| isotig54164 | 5.68861 | 0.881328 | sp\|Q8W493\|FNRL2_ARATH Ferredoxin--NADP reductase, leaf isozyme 2, chloroplastic OS=Arabidopsis thaliana GN=LFNR2 PE=1 SV=1 length=755 |
| isotig32509 | 5.68233 | 0.83016 | NO_ANNOT_AVAILABLE length=472 |
| isotig67211 | 4.54637 | 0.875214 | NO_ANNOT_AVAILABLE length=328 |
| contig12435 | 4.21804 | 0.865088 | sp\|Q9C7S5\|PSYR1_ARATH Tyrosine-sulfated glycopeptide receptor 1 OS=Arabidopsis thaliana GN=PSYR1 PE=2 SV=1 |
| isotig41714 | 4.02688 | 0.850466 | NO_ANNOT_AVAILABLE length=594 |
| isotig51070 | 3.32551 | 0.809949 | sp\|P08688\|ALB2_PEA Albumin-2 OS=Pisum sativum PE=2 SV=1 length=1046 |
| isotig69597 | 3.30448 | 0.805726 | sp\|P23993\|PSAL_HORVU Photosystem I reaction center subunit XI, chloroplastic OS=Hordeum vulgare GN=PSAL PE=1 SV=1 length=277 |
| isotig44109 | 3.26357 | 0.823301 | NO_ANNOT_AVAILABLE length=1013 |
| isotig03810 | 3.09523 | 0.818434 | sp\|P52428\|PSA1_ORYSJ Proteasome subunit alpha type-1 OS=Oryza sativa subsp. japonica GN=PAF1 PE=2 SV=1 length=1878 |
| isotig00022 | 2.24681 | 0.805938 | sp\|P37219\|ASR2_SOLLC Abscisic stress-ripening protein 2 OS=Solanum lycopersicum GN=ASR2 PE=4 SV=1 length=906 |
| isotig01248 | -2.59417 | 0.817086 | sp\|Q54PY7\|M2OM_DICDI Probable mitochondrial 2-oxoglutarate/malate carrier protein OS=Dictyostelium discoideum GN=ucpC PE=3 SV=1 length=1241 |
| isotig42388 | -3.03857 | 0.847184 | sp\|Q8TGM6\|TAR1_YEAST Protein TAR1 OS=Saccharomyces cerevisiae GN=TAR1 PE=2 SV=1 length=436 |
| contig05296 | -3.19388 | 0.836189 | NO_ANNOT_AVAILABLE |
| isotig25073 | -3.48137 | 0.800805 | sp\|Q05736\|PR1_ASPOF Pathogenesis-related protein 1 OS=Asparagus officinalis GN=PR1 PE=2 SV=1 length=658 |
| isotig64307 | -3.92632 | 0.882768 | NO_ANNOT_AVAILABLE length=386 |
| isotig20330 | -4.45443 | 0.829384 | NO_ANNOT_AVAILABLE length=251 |
| isotig65634 | -5.48198 | 0.815641 | sp\|Q8TGM6\|TAR1_YEAST Protein TAR1 OS=Saccharomyces cerevisiae GN=TAR1 PE=2 SV=1 length=352 |
| isotig00109 | -6.32310 | 0.912434 | sp\|Q6Z248\|HOX20_ORYSJ Homeobox-leucine zipper protein HOX20 OS=Oryza sativa subsp. japonica GN=HOX20 PE=2 SV=1 length=794 |
| isotig70862 | -6.41604 | 0.96207 | NO_ANNOT_AVAILABLE length=255 |
| isotig31718 | -7.61165 | 0.869547 | sp\|Q6CRQ9\|ISU1_KLULA Iron sulfur cluster assembly protein 1, mitochondrial OS=Kluyveromyces lactis GN=ISU1 PE=3 SV=1 length=1041 |

11:00

| **GeneID** | **log_2_Ratio** | **Q value** | Annotation |
| --- | --- | --- | --- |
| isotig42424 | 10.70750 | 0.99726 | NO_ANNOT_AVAILABLE length=621 |
| isotig21040 | 10.61840 | 0.98043 | sp\|P27489\|CB23_SOLLC Chlorophyll a-b binding protein 13, chloroplastic OS=Solanum lycopersicum GN=CAB13 PE=1 SV=1 length=1116 |
| isotig59762 | 9.28973 | 0.8645 | NO_ANNOT_AVAILABLE length=496 |
| contig12472 | 9.16045 | 0.85553 | sp\|Q0WR59\|Y5020_ARATH Probable inactive receptor kinase At5g10020 OS=Arabidopsis thaliana GN=At5g10020 PE=1 SV=2 |
| isotig67143 | 9.15861 | 0.85536 | sp\|P82413\|RK19_SPIOL 50S ribosomal protein L19, chloroplastic OS=Spinacia oleracea GN=RPL19 PE=1 SV=2 length=331 |
| isotig35527 | 9.03911 | 0.84687 | sp\|Q5RBU7\|PCP_PONAB Lysosomal Pro-X carboxypeptidase OS=Pongo abelii GN=PRCP PE=2 SV=1 length=827 |
| contig12490 | 8.98423 | 0.84268 | sp\|O22476\|BRI1_ARATH Protein BRASSINOSTEROID INSENSITIVE 1 OS=Arabidopsis thaliana GN=BRI1 PE=1 SV=1 |
| isotig70620 | 8.82588 | 0.83068 | NO_ANNOT_AVAILABLE length=262 |
| isotig21342 | 8.81554 | 0.82994 | NO_ANNOT_AVAILABLE length=975 |
| isotig65188 | 8.79531 | 0.95355 | sp\|Q9LRR5\|DRL21_ARATH Putative disease resistance protein At3g14460 OS=Arabidopsis thaliana GN=At3g14460 PE=2 SV=1 length=367 |
| isotig56650 | 7.86396 | 0.93628 | NO_ANNOT_AVAILABLE length=612 |
| isotig22997 | 6.81947 | 0.81595 | NO_ANNOT_AVAILABLE length=597 |
| isotig69058 | 5.68911 | 0.94401 | NO_ANNOT_AVAILABLE length=287 |
| isotig54164 | 5.62667 | 0.90437 | sp\|Q8W493\|FNRL2_ARATH Ferredoxin--NADP reductase, leaf isozyme 2, chloroplastic OS=Arabidopsis thaliana GN=LFNR2 PE=1 SV=1 length=755 |
| isotig12931 | 5.42915 | 0.82336 | NO_ANNOT_AVAILABLE length=827 |
| isotig35470 | 4.92354 | 0.85105 | sp\|P32295\|ARG7_PHAAU Indole-3-acetic acid-induced protein ARG7 OS=Phaseolus aureus GN=ARG7 PE=2 SV=1 length=896 |
| contig13817 | 4.62687 | 0.91964 | sp\|P83304\|LEC_PARPC Mannose/glucose-specific lectin (Fragment) OS=Parkia platycephala PE=1 SV=1 |
| isotig51070 | 3.64300 | 0.84951 | sp\|P08688\|ALB2_PEA Albumin-2 OS=Pisum sativum PE=2 SV=1 length=1046 |
| contig12435 | 3.45783 | 0.84466 | sp\|Q9C7S5\|PSYR1_ARATH Tyrosine-sulfated glycopeptide receptor 1 OS=Arabidopsis thaliana GN=PSYR1 PE=2 SV=1 |
| isotig57153 | 3.41742 | 0.81817 | NO_ANNOT_AVAILABLE length=587 |
| isotig23466 | 3.39809 | 0.8615 | sp\|P81713\|IBB3_WHEAT Bowman-Birk type trypsin inhibitor OS=Triticum aestivum PE=1 SV=1 length=573 |
| isotig56418 | 3.06845 | 0.83355 | sp\|P42390\|TRPA_MAIZE Indole-3-glycerol phosphate lyase, chloroplastic OS=Zea mays GN=BX1 PE=1 SV=2 length=623 |
| isotig35842 | 2.63892 | 0.81408 | NO_ANNOT_AVAILABLE length=893 |
| contig08982 | -2.55060 | 0.80435 | NO_ANNOT_AVAILABLE |
| isotig17755 | -2.82610 | 0.82194 | NO_ANNOT_AVAILABLE length=792 |
| isotig42388 | -3.11230 | 0.84164 | sp\|Q8TGM6\|TAR1_YEAST Protein TAR1 OS=Saccharomyces cerevisiae GN=TAR1 PE=2 SV=1 length=436 |
| isotig67552 | -3.83170 | 0.81367 | sp\|O06432\|TONB_NEIGO Protein tonB OS=Neisseria gonorrhoeae GN=tonB PE=3 SV=1 length=321 |
| isotig64307 | -4.32150 | 0.8973 | NO_ANNOT_AVAILABLE length=386 |
| isotig20330 | -5.64260 | 0.81846 | NO_ANNOT_AVAILABLE length=251 |
| isotig00109 | -7.63090 | 0.92827 | sp\|Q6Z248\|HOX20_ORYSJ Homeobox-leucine zipper protein HOX20 OS=Oryza sativa subsp. japonica GN=HOX20 PE=2 SV=1 length=794 |
| isotig31718 | -7.91160 | 0.83893 | sp\|Q6CRQ9\|ISU1_KLULA Iron sulfur cluster assembly protein 1, mitochondrial OS=Kluyveromyces lactis GN=ISU1 PE=3 SV=1 length=1041 |

15:00

| **GeneID** | **log_2_Ratio** | **Q value** | **Annotation** |
| --- | --- | --- | --- |
| isotig56650 | 8.92928 | 0.95948 | NO_ANNOT_AVAILABLE length=612 |
| isotig65143 | 5.10297 | 0.94788 | sp\|Q9SBK6\|JMT_BRARP Jasmonate O-methyltransferase OS=Brassica rapa subsp. pekinensis GN=JMT PE=1 SV=1 length=369 |
| isotig54164 | 2.82175 | 0.83754 | sp\|Q8W493\|FNRL2_ARATH Ferredoxin--NADP reductase, leaf isozyme 2, chloroplastic OS=Arabidopsis thaliana GN=LFNR2 PE=1 SV=1 length=755 |
| isotig47569 | 2.43760 | 0.8248 | NO_ANNOT_AVAILABLE length=1716 |
| isotig48778 | 2.18561 | 0.81394 | sp\|Q93X23\|MYRS_QUEIL Myrcene synthase, chloroplastic OS=Quercus ilex PE=1 SV=1 length=1416 |
| isotig69058 | 2.16980 | 0.80161 | NO_ANNOT_AVAILABLE length=287 |
| isotig61986 | 2.12960 | 0.80715 | sp\|Q40577\|5EAS_TOBAC Aristolochene synthase OS=Nicotiana tabacum GN=EAS3 PE=1 SV=3 length=435 |
| isotig45092 | -2.21890 | 0.8118 | NO_ANNOT_AVAILABLE length=301 |
| contig08982 | -2.42530 | 0.81017 | NO_ANNOT_AVAILABLE |
| isotig70360 | -3.14790 | 0.86819 | NO_ANNOT_AVAILABLE length=266 |
| isotig56670 | -3.27250 | 0.87328 | sp\|P55857\|SMT3_ORYSJ Ubiquitin-like protein SMT3 OS=Oryza sativa subsp. japonica GN=SMT3 PE=3 SV=1 length=610 |
| isotig00109 | -7.86040 | 0.94296 | sp\|Q6Z248\|HOX20_ORYSJ Homeobox-leucine zipper protein HOX20 OS=Oryza sativa subsp. japonica GN=HOX20 PE=2 SV=1 length=794 |

19:00

| **GeneID** | **log_2_Ratio** | **Q value** | **Annotation** |
| --- | --- | --- | --- |
| isotig42424 | 12.95720 | 0.99901 | NO_ANNOT_AVAILABLE length=621 |
| isotig65188 | 11.03150 | 0.96432 | sp\|Q9LRR5\|DRL21_ARATH Putative disease resistance protein At3g14460 OS=Arabidopsis thaliana GN=At3g14460 PE=2 SV=1 length=367 |
| isotig70620 | 10.27000 | 0.93969 | NO_ANNOT_AVAILABLE length=262 |
| isotig63598 | 9.87196 | 0.9218 | NO_ANNOT_AVAILABLE length=401 |
| isotig59762 | 9.80279 | 0.91832 | NO_ANNOT_AVAILABLE length=496 |
| isotig22997 | 9.70154 | 0.91305 | NO_ANNOT_AVAILABLE length=597 |
| contig12472 | 9.19908 | 0.88248 | sp\|Q0WR59\|Y5020_ARATH Probable inactive receptor kinase At5g10020 OS=Arabidopsis thaliana GN=At5g10020 PE=1 SV=2 |
| contig12490 | 9.16916 | 0.88045 | sp\|O22476\|BRI1_ARATH Protein BRASSINOSTEROID INSENSITIVE 1 OS=Arabidopsis thaliana GN=BRI1 PE=1 SV=1 |
| isotig60716 | 8.67909 | 0.84336 | NO_ANNOT_AVAILABLE length=470 |
| isotig21040 | 8.65554 | 0.84135 | sp\|P27489\|CB23_SOLLC Chlorophyll a-b binding protein 13, chloroplastic OS=Solanum lycopersicum GN=CAB13 PE=1 SV=1 length=1116 |
| isotig56650 | 8.64430 | 0.94573 | NO_ANNOT_AVAILABLE length=612 |
| isotig21342 | 8.30340 | 0.87613 | NO_ANNOT_AVAILABLE length=975 |
| isotig35527 | 8.28391 | 0.80838 | sp\|Q5RBU7\|PCP_PONAB Lysosomal Pro-X carboxypeptidase OS=Pongo abelii GN=PRCP PE=2 SV=1 length=827 |
| isotig07740 | 7.90131 | 0.91822 | NO_ANNOT_AVAILABLE length=1005 |
| isotig67143 | 7.82878 | 0.90614 | sp\|P82413\|RK19_SPIOL 50S ribosomal protein L19, chloroplastic OS=Spinacia oleracea GN=RPL19 PE=1 SV=2 length=331 |
| isotig69058 | 6.03532 | 0.93208 | NO_ANNOT_AVAILABLE length=287 |
| isotig23475 | 5.91369 | 0.84766 | sp\|Q76MV0\|H32_TOBAC Histone H3.2 OS=Nicotiana tabacum GN=B34 PE=1 SV=1 length=747 |
| isotig46207 | 5.64968 | 0.86002 | NO_ANNOT_AVAILABLE length=2312 |
| isotig32509 | 5.49801 | 0.85338 | NO_ANNOT_AVAILABLE length=472 |
| isotig12931 | 5.26357 | 0.86294 | NO_ANNOT_AVAILABLE length=827 |
| isotig54164 | 5.13940 | 0.94484 | sp\|Q8W493\|FNRL2_ARATH Ferredoxin--NADP reductase, leaf isozyme 2, chloroplastic OS=Arabidopsis thaliana GN=LFNR2 PE=1 SV=1 length=755 |
| contig12469 | 5.13192 | 0.85426 | NO_ANNOT_AVAILABLE |
| isotig35470 | 4.76626 | 0.87637 | sp\|P32295\|ARG7_PHAAU Indole-3-acetic acid-induced protein ARG7 OS=Phaseolus aureus GN=ARG7 PE=2 SV=1 length=896 |
| contig12435 | 4.39121 | 0.90344 | sp\|Q9C7S5\|PSYR1_ARATH Tyrosine-sulfated glycopeptide receptor 1 OS=Arabidopsis thaliana GN=PSYR1 PE=2 SV=1 |
| isotig00714 | 4.23784 | 0.81301 | NO_ANNOT_AVAILABLE length=586 |
| isotig42732 | 4.12807 | 0.8163 | sp\|Q8W493\|FNRL2_ARATH Ferredoxin--NADP reductase, leaf isozyme 2, chloroplastic OS=Arabidopsis thaliana GN=LFNR2 PE=1 SV=1 length=433 |
| isotig44109 | 3.96814 | 0.87897 | NO_ANNOT_AVAILABLE length=1013 |
| isotig41714 | 3.84693 | 0.80954 | NO_ANNOT_AVAILABLE length=594 |
| isotig59805 | 3.84637 | 0.82091 | sp\|Q55E65\|Y0496_DICDI Putative uncharacterized protein DDB_G0270496 OS=Dictyostelium discoideum GN=DDB_G0270496 PE=3 SV=1 length=495 |
| contig05306 | 3.66112 | 0.82059 | NO_ANNOT_AVAILABLE |
| isotig58569 | 3.47244 | 0.82773 | sp\|Q9FKW6\|FNRL1_ARATH Ferredoxin--NADP reductase, leaf isozyme 1, chloroplastic OS=Arabidopsis thaliana GN=LFNR1 PE=1 SV=1 length=532 |
| isotig39820 | 3.45482 | 0.85783 | NO_ANNOT_AVAILABLE length=867 |
| isotig67211 | 3.29436 | 0.85201 | NO_ANNOT_AVAILABLE length=328 |
| isotig40959 | 2.99265 | 0.81039 | NO_ANNOT_AVAILABLE length=726 |
| isotig03810 | 2.98812 | 0.83738 | sp\|P52428\|PSA1_ORYSJ Proteasome subunit alpha type-1 OS=Oryza sativa subsp. japonica GN=PAF1 PE=2 SV=1 length=1878 |
| isotig23017 | 2.88530 | 0.82181 | NO_ANNOT_AVAILABLE length=618 |
| isotig69597 | 2.76769 | 0.83335 | sp\|P23993\|PSAL_HORVU Photosystem I reaction center subunit XI, chloroplastic OS=Hordeum vulgare GN=PSAL PE=1 SV=1 length=277 |
| isotig19213 | 2.52698 | 0.84855 | sp\|Q9SQL5\|SODC_ANACO Superoxide dismutase [Cu-Zn] OS=Ananas comosus GN=SOD1 PE=2 SV=1 length=918 |
| isotig28775 | 2.50050 | 0.8214 | NO_ANNOT_AVAILABLE length=636 |
| isotig65178 | 2.49373 | 0.81888 | NO_ANNOT_AVAILABLE length=367 |
| isotig35548 | 2.34628 | 0.82038 | sp\|P49211\|RL321_ARATH 60S ribosomal protein L32-1 OS=Arabidopsis thaliana GN=RPL32A PE=2 SV=2 length=795 |
| isotig30968 | 2.19553 | 0.81177 | sp\|Q5XHZ9\|TRP13_RAT Thyroid receptor-interacting protein 13 OS=Rattus norvegicus GN=Trip13 PE=2 SV=1 length=1401 |
| isotig00682 | 2.06607 | 0.80415 | sp\|P55852\|SMT3_ARATH Ubiquitin-like protein SMT3 OS=Arabidopsis thaliana GN=SMT3 PE=1 SV=2 length=682 |
| isotig01797 | -1.72160 | 0.80154 | sp\|P38419\|LOXC1_ORYSJ Lipoxygenase 7, chloroplastic OS=Oryza sativa subsp. japonica GN=CM-LOX1 PE=2 SV=2 length=3039 |
| contig08913 | -1.94860 | 0.82169 | sp\|Q942D4\|BURP3_ORYSJ BURP domain-containing protein 3 OS=Oryza sativa subsp. japonica GN=BURP3 PE=2 SV=1 |
| isotig23468 | -1.95600 | 0.82135 | sp\|P81713\|IBB3_WHEAT Bowman-Birk type trypsin inhibitor OS=Triticum aestivum PE=1 SV=1 length=573 |
| isotig57870 | -2.00640 | 0.80077 | sp\|Q95JC9\|PRP_PIG Basic proline-rich protein OS=Sus scrofa PE=1 SV=2 length=558 |
| isotig00023 | -2.02860 | 0.82651 | sp\|P37219\|ASR2_SOLLC Abscisic stress-ripening protein 2 OS=Solanum lycopersicum GN=ASR2 PE=4 SV=1 length=906 |
| contig72097 | -2.10330 | 0.80452 | sp\|Q6H543\|IAA7_ORYSJ Auxin-responsive protein IAA7 OS=Oryza sativa subsp. japonica GN=IAA7 PE=2 SV=1 |
| isotig70360 | -2.15520 | 0.83022 | NO_ANNOT_AVAILABLE length=266 |
| isotig10327 | -2.15720 | 0.80198 | NO_ANNOT_AVAILABLE length=249 |
| isotig16579 | -2.24970 | 0.80799 | NO_ANNOT_AVAILABLE length=428 |
| isotig55862 | -2.32610 | 0.82629 | NO_ANNOT_AVAILABLE length=653 |
| isotig10324 | -2.35370 | 0.83884 | NO_ANNOT_AVAILABLE length=1448 |
| isotig14822 | -2.42610 | 0.82957 | NO_ANNOT_AVAILABLE length=1222 |
| contig72101 | -2.44330 | 0.80274 | NO_ANNOT_AVAILABLE |
| isotig50136 | -2.47770 | 0.82461 | sp\|Q96502\|COL2_ARATH Zinc finger protein CONSTANS-LIKE 2 OS=Arabidopsis thaliana GN=COL2 PE=1 SV=1 length=1177 |
| isotig42507 | -2.61890 | 0.85711 | sp\|Q0DKW8\|LTI6B_ORYSJ Hydrophobic protein LTI6B OS=Oryza sativa subsp. japonica GN=LTI6B PE=2 SV=1 length=707 |
| isotig56587 | -2.66240 | 0.83876 | NO_ANNOT_AVAILABLE length=610 |
| isotig53311 | -2.66420 | 0.84425 | NO_ANNOT_AVAILABLE length=820 |
| isotig42387 | -2.83940 | 0.83706 | sp\|Q8TGM6\|TAR1_YEAST Protein TAR1 OS=Saccharomyces cerevisiae GN=TAR1 PE=2 SV=1 length=472 |
| isotig65070 | -2.89930 | 0.84493 | NO_ANNOT_AVAILABLE length=370 |
| isotig49343 | -3.00810 | 0.87835 | sp\|O49255\|NAC29_ARATH NAC domain-containing protein 29 OS=Arabidopsis thaliana GN=NAC029 PE=2 SV=1 length=1309 |
| isotig56670 | -3.08530 | 0.88385 | sp\|P55857\|SMT3_ORYSJ Ubiquitin-like protein SMT3 OS=Oryza sativa subsp. japonica GN=SMT3 PE=3 SV=1 length=610 |
| contig08982 | -3.08860 | 0.85001 | NO_ANNOT_AVAILABLE |
| contig05296 | -3.13410 | 0.86331 | NO_ANNOT_AVAILABLE |
| isotig71259 | -3.41360 | 0.80746 | NO_ANNOT_AVAILABLE length=252 |
| isotig58953 | -3.77240 | 0.90596 | sp\|Q6K8Z4\|FH7_ORYSJ Formin-like protein 7 OS=Oryza sativa subsp. japonica GN=FH7 PE=2 SV=2 length=521 |
| isotig42388 | -3.80460 | 0.90762 | sp\|Q8TGM6\|TAR1_YEAST Protein TAR1 OS=Saccharomyces cerevisiae GN=TAR1 PE=2 SV=1 length=436 |
| isotig68281 | -3.89710 | 0.81831 | NO_ANNOT_AVAILABLE length=305 |
| contig80253 | -4.11490 | 0.83565 | sp\|Q2R4Z4\|DHR21_ORYSJ Water stress-inducible protein Rab21 OS=Oryza sativa subsp. japonica GN=RAB21 PE=2 SV=1 |
| isotig45273 | -4.13590 | 0.89979 | NO_ANNOT_AVAILABLE length=619 |
| isotig01248 | -4.32940 | 0.92294 | sp\|Q54PY7\|M2OM_DICDI Probable mitochondrial 2-oxoglutarate/malate carrier protein OS=Dictyostelium discoideum GN=ucpC PE=3 SV=1 length=1241 |
| isotig63333 | -4.80880 | 0.81362 | NO_ANNOT_AVAILABLE length=407 |
| isotig53239 | -4.83620 | 0.82741 | sp\|Q84MC2\|Y5195_ARATH UPF0717 protein At5g11950 OS=Arabidopsis thaliana GN=At5g11950 PE=1 SV=1 length=828 |
| isotig64307 | -5.00190 | 0.95135 | NO_ANNOT_AVAILABLE length=386 |
| isotig60482 | -5.05380 | 0.8843 | NO_ANNOT_AVAILABLE length=476 |
| isotig60334 | -5.74490 | 0.91433 | NO_ANNOT_AVAILABLE length=481 |
| isotig56002 | -5.96880 | 0.92155 | NO_ANNOT_AVAILABLE length=645 |
| isotig56852 | -6.04590 | 0.89373 | sp\|O04017\|NAC98_ARATH Protein CUP-SHAPED COTYLEDON 2 OS=Arabidopsis thaliana GN=NAC098 PE=1 SV=1 length=603 |
| isotig41992 | -6.05480 | 0.84089 | NO_ANNOT_AVAILABLE length=639 |
| isotig17341 | -6.97520 | 0.98682 | sp\|P31752\|ASNS_ASPOF Asparagine synthetase [glutamine-hydrolyzing] OS=Asparagus officinalis PE=2 SV=2 length=1929 |
| isotig31718 | -7.20030 | 0.81908 | sp\|Q6CRQ9\|ISU1_KLULA Iron sulfur cluster assembly protein 1, mitochondrial OS=Kluyveromyces lactis GN=ISU1 PE=3 SV=1 length=1041 |
| isotig00109 | -7.47410 | 0.94159 | sp\|Q6Z248\|HOX20_ORYSJ Homeobox-leucine zipper protein HOX20 OS=Oryza sativa subsp. japonica GN=HOX20 PE=2 SV=1 length=794 |
| isotig21282 | -7.83410 | 0.82433 | sp\|O66601\|GUAA_AQUAE GMP synthase [glutamine-hydrolyzing] OS=Aquifex aeolicus GN=guaA PE=3 SV=1 length=2062 |
| isotig53829 | -8.17380 | 0.98229 | NO_ANNOT_AVAILABLE length=779 |
| isotig52156 | -8.22950 | 0.95119 | sp\|P09444\|LEA34_GOSHI Late embryogenesis abundant protein D-34 OS=Gossypium hirsutum PE=4 SV=1 length=930 |
| isotig34356 | -8.29520 | 0.80926 | NO_ANNOT_AVAILABLE length=860 |
| isotig56846 | -9.92640 | 0.92449 | NO_ANNOT_AVAILABLE length=604 |

07:00

| **GeneID** | **log_2_Ratio** | **Q value** | **Annotation** |
| --- | --- | --- | --- |
| isotig21342 | 9.46548 | 0.89387 | NO_ANNOT_AVAILABLE length=975 |
| isotig59762 | 9.29138 | 0.88318 | NO_ANNOT_AVAILABLE length=496 |
| isotig42424 | 9.26365 | 0.98698 | NO_ANNOT_AVAILABLE length=621 |
| isotig35527 | 9.00252 | 0.91416 | sp\|Q5RBU7\|PCP_PONAB Lysosomal Pro-X carboxypeptidase OS=Pongo abelii GN=PRCP PE=2 SV=1 length=827 |
| isotig22997 | 8.85308 | 0.85302 | NO_ANNOT_AVAILABLE length=597 |
| isotig65188 | 8.83801 | 0.97012 | sp\|Q9LRR5\|DRL21_ARATH Putative disease resistance protein At3g14460 OS=Arabidopsis thaliana GN=At3g14460 PE=2 SV=1 length=367 |
| isotig67143 | 7.12045 | 0.84326 | sp\|P82413\|RK19_SPIOL 50S ribosomal protein L19, chloroplastic OS=Spinacia oleracea GN=RPL19 PE=1 SV=2 length=331 |
| isotig56650 | 6.21729 | 0.84583 | NO_ANNOT_AVAILABLE length=612 |
| isotig69058 | 5.57308 | 0.9385 | NO_ANNOT_AVAILABLE length=287 |
| isotig41714 | 4.55139 | 0.80965 | NO_ANNOT_AVAILABLE length=594 |
| isotig58710 | 4.08795 | 0.80675 | NO_ANNOT_AVAILABLE length=531 |
| isotig67211 | 3.94943 | 0.84745 | NO_ANNOT_AVAILABLE length=328 |
| contig12435 | 3.78165 | 0.84694 | sp\|Q9C7S5\|PSYR1_ARATH Tyrosine-sulfated glycopeptide receptor 1 OS=Arabidopsis thaliana GN=PSYR1 PE=2 SV=1 |
| isotig44109 | 3.11712 | 0.82758 | NO_ANNOT_AVAILABLE length=1013 |
| isotig03810 | 2.97834 | 0.81221 | sp\|P52428\|PSA1_ORYSJ Proteasome subunit alpha type-1 OS=Oryza sativa subsp. japonica GN=PAF1 PE=2 SV=1 length=1878 |
| isotig35548 | 2.75437 | 0.81544 | sp\|P49211\|RL321_ARATH 60S ribosomal protein L32-1 OS=Arabidopsis thaliana GN=RPL32A PE=2 SV=2 length=795 |
| isotig19213 | 2.24773 | 0.80453 | sp\|Q9SQL5\|SODC_ANACO Superoxide dismutase [Cu-Zn] OS=Ananas comosus GN=SOD1 PE=2 SV=1 length=918 |
| contig16788 | 2.10553 | 0.80541 | NO_ANNOT_AVAILABLE |
| contig08913 | -1.98690 | 0.80378 | sp\|Q942D4\|BURP3_ORYSJ BURP domain-containing protein 3 OS=Oryza sativa subsp. japonica GN=BURP3 PE=2 SV=1 |
| isotig56670 | -2.17220 | 0.81513 | sp\|P55857\|SMT3_ORYSJ Ubiquitin-like protein SMT3 OS=Oryza sativa subsp. japonica GN=SMT3 PE=3 SV=1 length=610 |
| isotig01248 | -2.49520 | 0.8264 | sp\|Q54PY7\|M2OM_DICDI Probable mitochondrial 2-oxoglutarate/malate carrier protein OS=Dictyostelium discoideum GN=ucpC PE=3 SV=1 length=1241 |
| contig08982 | -3.53760 | 0.88537 | NO_ANNOT_AVAILABLE |
| isotig68281 | -3.65290 | 0.80208 | NO_ANNOT_AVAILABLE length=305 |
| isotig20330 | -5.55040 | 0.83452 | NO_ANNOT_AVAILABLE length=251 |
| isotig00109 | -7.90910 | 0.91706 | sp\|Q6Z248\|HOX20_ORYSJ Homeobox-leucine zipper protein HOX20 OS=Oryza sativa subsp. japonica GN=HOX20 PE=2 SV=1 length=794 |
| isotig31718 | -8.15780 | 0.8839 | sp\|Q6CRQ9\|ISU1_KLULA Iron sulfur cluster assembly protein 1, mitochondrial OS=Kluyveromyces lactis GN=ISU1 PE=3 SV=1 length=1041 |
| isotig21282 | -8.47180 | 0.82314 | sp\|O66601\|GUAA_AQUAE GMP synthase [glutamine-hydrolyzing] OS=Aquifex aeolicus GN=guaA PE=3 SV=1 length=2062 |
