# Supplementary material for: Diurnal biomarkers reveal key photosynthetic genes associated with increased oil palm yield
Source: PLoS One. 2019 Mar 11;14(3):e0213591. doi: 10.1371/journal.pone.0213591 (PMC6411157; doi:10.1371/journal.pone.0213591)
Supplement: S6 Table — (DOCX) [file pone.0213591.s009.docx]

**Supplementary table 6**

Overlapping or unique differentially expressed genes across different time points.

| (A) 7:00 |  |
| --- | --- |
| Isotig | Annotation |
| isotig72193 | NO_ANNOT_AVAILABLE length=229 |
| isotig00022 | sp\|P37219\|ASR2_SOLLC Abscisic stress-ripening protein 2 OS=Solanum lycopersicum GN=ASR2 PE=4 SV=1 length=906 |
| isotig25073 | sp\|Q05736\|PR1_ASPOF Pathogenesis-related protein 1 OS=Asparagus officinalis GN=PR1 PE=2 SV=1 length=658 |
| isotig65634 | sp\|Q8TGM6\|TAR1_YEAST Protein TAR1 OS=Saccharomyces cerevisiae GN=TAR1 PE=2 SV=1 length=352 |
| isotig70862 | NO_ANNOT_AVAILABLE length=255 |
|  |  |
| (B) 11:00 |  |
| Isotig | Annotation |
| contig13817 | sp\|P83304\|LEC_PARPC Mannose/glucose-specific lectin (Fragment) OS=Parkia platycephala PE=1 SV=1 |
| isotig57153 | NO_ANNOT_AVAILABLE length=587 |
| isotig23466 | sp\|P81713\|IBB3_WHEAT Bowman-Birk type trypsin inhibitor OS=Triticum aestivum PE=1 SV=1 length=573 |
| isotig56418 | sp\|P42390\|TRPA_MAIZE Indole-3-glycerol phosphate lyase, chloroplastic OS=Zea mays GN=BX1 PE=1 SV=2 length=623 |
| isotig35842 | NO_ANNOT_AVAILABLE length=893 |
| isotig17755 | NO_ANNOT_AVAILABLE length=792 |
| isotig67552 | sp\|O06432\|TONB_NEIGO Protein tonB OS=Neisseria gonorrhoeae GN=tonB PE=3 SV=1 length=321 |
|  |  |
| (C) 15:00 |  |
| isotig | Annotation |
| isotig65143 | sp\|Q9SBK6\|JMT_BRARP Jasmonate O-methyltransferase OS=Brassica rapa subsp. pekinensis GN=JMT PE=1 SV=1 length=369 |
| isotig47569 | NO_ANNOT_AVAILABLE length=1716 |
| isotig48778 | sp\|Q93X23\|MYRS_QUEIL Myrcene synthase, chloroplastic OS=Quercus ilex PE=1 SV=1 length=1416 |
| isotig61986 | sp\|Q40577\|5EAS_TOBAC Aristolochene synthase OS=Nicotiana tabacum GN=EAS3 PE=1 SV=3 length=435 |
| isotig45092 | NO_ANNOT_AVAILABLE length=301 |
|  |  |
| (D) 19:00 |  |
| isotig | Annotation |
| contig12469 | NO_ANNOT_AVAILABLE |
| contig05306 | NO_ANNOT_AVAILABLE |
| contig72101 | NO_ANNOT_AVAILABLE |
| isotig07740 | NO_ANNOT_AVAILABLE length=1005 |
| isotig14822 | NO_ANNOT_AVAILABLE length=1222 |
| isotig10324 | NO_ANNOT_AVAILABLE length=1448 |
| isotig46207 | NO_ANNOT_AVAILABLE length=2312 |
| isotig10327 | NO_ANNOT_AVAILABLE length=249 |
| isotig71259 | NO_ANNOT_AVAILABLE length=252 |
| isotig65178 | NO_ANNOT_AVAILABLE length=367 |
| isotig65070 | NO_ANNOT_AVAILABLE length=370 |
| isotig63598 | NO_ANNOT_AVAILABLE length=401 |
| isotig63333 | NO_ANNOT_AVAILABLE length=407 |
| isotig16579 | NO_ANNOT_AVAILABLE length=428 |
| isotig60716 | NO_ANNOT_AVAILABLE length=470 |
| isotig60482 | NO_ANNOT_AVAILABLE length=476 |
| isotig60334 | NO_ANNOT_AVAILABLE length=481 |
| isotig00714 | NO_ANNOT_AVAILABLE length=586 |
| isotig56846 | NO_ANNOT_AVAILABLE length=604 |
| isotig56587 | NO_ANNOT_AVAILABLE length=610 |
| isotig23017 | NO_ANNOT_AVAILABLE length=618 |
| isotig45273 | NO_ANNOT_AVAILABLE length=619 |
| isotig28775 | NO_ANNOT_AVAILABLE length=636 |
| isotig41992 | NO_ANNOT_AVAILABLE length=639 |
| isotig56002 | NO_ANNOT_AVAILABLE length=645 |
| isotig55862 | NO_ANNOT_AVAILABLE length=653 |
| isotig40959 | NO_ANNOT_AVAILABLE length=726 |
| isotig53829 | NO_ANNOT_AVAILABLE length=779 |
| isotig53311 | NO_ANNOT_AVAILABLE length=820 |
| isotig34356 | NO_ANNOT_AVAILABLE length=860 |
| isotig39820 | NO_ANNOT_AVAILABLE length=867 |
| isotig56852 | sp\|O04017\|NAC98_ARATH Protein CUP-SHAPED COTYLEDON 2 OS=Arabidopsis thaliana GN=NAC098 PE=1 SV=1 length=603 |
| isotig49343 | sp\|O49255\|NAC29_ARATH NAC domain-containing protein 29 OS=Arabidopsis thaliana GN=NAC029 PE=2 SV=1 length=1309 |
| isotig52156 | sp\|P09444\|LEA34_GOSHI Late embryogenesis abundant protein D-34 OS=Gossypium hirsutum PE=4 SV=1 length=930 |
| isotig17341 | sp\|P31752\|ASNS_ASPOF Asparagine synthetase [glutamine-hydrolyzing] OS=Asparagus officinalis PE=2 SV=2 length=1929 |
| isotig00023 | sp\|P37219\|ASR2_SOLLC Abscisic stress-ripening protein 2 OS=Solanum lycopersicum GN=ASR2 PE=4 SV=1 length=906 |
| isotig01797 | sp\|P38419\|LOXC1_ORYSJ Lipoxygenase 7, chloroplastic OS=Oryza sativa subsp. japonica GN=CM-LOX1 PE=2 SV=2 length=3039 |
| isotig00682 | sp\|P55852\|SMT3_ARATH Ubiquitin-like protein SMT3 OS=Arabidopsis thaliana GN=SMT3 PE=1 SV=2 length=682 |
| isotig23468 | sp\|P81713\|IBB3_WHEAT Bowman-Birk type trypsin inhibitor OS=Triticum aestivum PE=1 SV=1 length=573 |
| isotig42507 | sp\|Q0DKW8\|LTI6B_ORYSJ Hydrophobic protein LTI6B OS=Oryza sativa subsp. japonica GN=LTI6B PE=2 SV=1 length=707 |
| contig80253 | sp\|Q2R4Z4\|DHR21_ORYSJ Water stress-inducible protein Rab21 OS=Oryza sativa subsp. japonica GN=RAB21 PE=2 SV=1 |
| isotig59805 | sp\|Q55E65\|Y0496_DICDI Putative uncharacterized protein DDB_G0270496 OS=Dictyostelium discoideum GN=DDB_G0270496 PE=3 SV=1 length=495 |
| isotig30968 | sp\|Q5XHZ9\|TRP13_RAT Thyroid receptor-interacting protein 13 OS=Rattus norvegicus GN=Trip13 PE=2 SV=1 length=1401 |
| contig72097 | sp\|Q6H543\|IAA7_ORYSJ Auxin-responsive protein IAA7 OS=Oryza sativa subsp. japonica GN=IAA7 PE=2 SV=1 |
| isotig58953 | sp\|Q6K8Z4\|FH7_ORYSJ Formin-like protein 7 OS=Oryza sativa subsp. japonica GN=FH7 PE=2 SV=2 length=521 |
| isotig23475 | sp\|Q76MV0\|H32_TOBAC Histone H3.2 OS=Nicotiana tabacum GN=B34 PE=1 SV=1 length=747 |
| isotig53239 | sp\|Q84MC2\|Y5195_ARATH UPF0717 protein At5g11950 OS=Arabidopsis thaliana GN=At5g11950 PE=1 SV=1 length=828 |
| isotig42387 | sp\|Q8TGM6\|TAR1_YEAST Protein TAR1 OS=Saccharomyces cerevisiae GN=TAR1 PE=2 SV=1 length=472 |
| isotig42732 | sp\|Q8W493\|FNRL2_ARATH Ferredoxin--NADP reductase, leaf isozyme 2, chloroplastic OS=Arabidopsis thaliana GN=LFNR2 PE=1 SV=1 length=433 |
| isotig57870 | sp\|Q95JC9\|PRP_PIG Basic proline-rich protein OS=Sus scrofa PE=1 SV=2 length=558 |
| isotig50136 | sp\|Q96502\|COL2_ARATH Zinc finger protein CONSTANS-LIKE 2 OS=Arabidopsis thaliana GN=COL2 PE=1 SV=1 length=1177 |
| isotig58569 | sp\|Q9FKW6\|FNRL1_ARATH Ferredoxin--NADP reductase, leaf isozyme 1, chloroplastic OS=Arabidopsis thaliana GN=LFNR1 PE=1 SV=1 length=532 |
|  |  |
| (E) 7:00-Next day | |
| isotig | Annotation |
| isotig58710 | NO_ANNOT_AVAILABLE length=531 |
| contig16788 | NO_ANNOT_AVAILABLE |
|  |  |
| (F) 7:00 Vs 11:00 | |
| isotig | Annotation |
| isotig51070 | sp\|P08688\|ALB2_PEA Albumin-2 OS=Pisum sativum PE=2 SV=1 length=1046 |
|  |  |
| (G) 7:00 Vs 19:00 | |
| isotig | Annotation |
| isotig32509 | NO_ANNOT_AVAILABLE length=472 |
| isotig69597 | sp\|P23993\|PSAL_HORVU Photosystem I reaction center subunit XI, chloroplastic OS=Hordeum vulgare GN=PSAL PE=1 SV=1 length=277 |
| contig05296 | NO_ANNOT_AVAILABLE |
|  |  |
| (H) 11:00 Vs 19:00 | |
| isotig | Annotation |
| contig12472 | sp\|Q0WR59\|Y5020_ARATH Probable inactive receptor kinase At5g10020 OS=Arabidopsis thaliana GN=At5g10020 PE=1 SV=2 |
| contig12490 | sp\|O22476\|BRI1_ARATH Protein BRASSINOSTEROID INSENSITIVE 1 OS=Arabidopsis thaliana GN=BRI1 PE=1 SV=1 |
| isotig12931 | NO_ANNOT_AVAILABLE length=827 |
| isotig35470 | sp\|P32295\|ARG7_PHAAU Indole-3-acetic acid-induced protein ARG7 OS=Phaseolus aureus GN=ARG7 PE=2 SV=1 length=896 |
|  |  |
| (I) 15:00 Vs 19:00 | |
| isotig | Annotation |
| isotig70360 | NO_ANNOT_AVAILABLE length=266 |
|  |  |
| (J) 19:00 Vs 7:00-Next Day | |
| isotig | Annotation |
| isotig19213 | sp\|Q9SQL5\|SODC_ANACO Superoxide dismutase [Cu-Zn] OS=Ananas comosus GN=SOD1 PE=2 SV=1 length=918 |
| isotig35548 | sp\|P49211\|RL321_ARATH 60S ribosomal protein L32-1 OS=Arabidopsis thaliana GN=RPL32A PE=2 SV=2 length=795 |
| contig08913 | sp\|Q942D4\|BURP3_ORYSJ BURP domain-containing protein 3 OS=Oryza sativa subsp. japonica GN=BURP3 PE=2 SV=1 |
| isotig68281 | NO_ANNOT_AVAILABLE length=305 |
| isotig21282 | sp\|O66601\|GUAA_AQUAE GMP synthase [glutamine-hydrolyzing] OS=Aquifex aeolicus GN=guaA PE=3 SV=1 length=2062 |
|  |  |
| (K) 7:00 Vs 11:00 Vs 19:00 | |
| isotig | Annotation |
| isotig70620 | NO_ANNOT_AVAILABLE length=262 |
| isotig21040 | sp\|P27489\|CB23_SOLLC Chlorophyll a-b binding protein 13, chloroplastic OS=Solanum lycopersicum GN=CAB13 PE=1 SV=1 length=1116 |
| isotig42388 | sp\|Q8TGM6\|TAR1_YEAST Protein TAR1 OS=Saccharomyces cerevisiae GN=TAR1 PE=2 SV=1 length=436 |
| isotig64307 | NO_ANNOT_AVAILABLE length=386 |
|  |  |
| (L) 7:00 Vs 11:00 Vs 7:00-Next day | |
| isotig | Annotation |
| isotig20330 | NO_ANNOT_AVAILABLE length=251 |
|  |  |
| (M) 7:00 Vs 19:00 Vs 7:00-Next day | |
| isotig | Annotation |
| isotig67211 | NO_ANNOT_AVAILABLE length=328 |
| isotig41714 | NO_ANNOT_AVAILABLE length=594 |
| isotig44109 | NO_ANNOT_AVAILABLE length=1013 |
| isotig03810 | sp\|P52428\|PSA1_ORYSJ Proteasome subunit alpha type-1 OS=Oryza sativa subsp. japonica GN=PAF1 PE=2 SV=1 length=1878 |
| isotig01248 | sp\|Q54PY7\|M2OM_DICDI Probable mitochondrial 2-oxoglutarate/malate carrier protein OS=Dictyostelium discoideum GN=ucpC PE=3 SV=1 length=1241 |
|  |  |
| (N) 7:00 Vs 11:00 Vs 19:00 Vs 7:00-Next day | |
| isotig | Annotation |
| isotig65188 | sp\|Q9LRR5\|DRL21_ARATH Putative disease resistance protein At3g14460 OS=Arabidopsis thaliana GN=At3g14460 PE=2 SV=1 length=367 |
| isotig42424 | NO_ANNOT_AVAILABLE length=621 |
| isotig35527 | sp\|Q5RBU7\|PCP_PONAB Lysosomal Pro-X carboxypeptidase OS=Pongo abelii GN=PRCP PE=2 SV=1 length=827 |
| isotig22997 | NO_ANNOT_AVAILABLE length=597 |
| isotig59762 | NO_ANNOT_AVAILABLE length=496 |
| isotig67143 | sp\|P82413\|RK19_SPIOL 50S ribosomal protein L19, chloroplastic OS=Spinacia oleracea GN=RPL19 PE=1 SV=2 length=331 |
| isotig21342 | NO_ANNOT_AVAILABLE length=975 |
| contig12435 | sp\|Q9C7S5\|PSYR1_ARATH Tyrosine-sulfated glycopeptide receptor 1 OS=Arabidopsis thaliana GN=PSYR1 PE=2 SV=1 |
| isotig31718 | sp\|Q6CRQ9\|ISU1_KLULA Iron sulfur cluster assembly protein 1, mitochondrial OS=Kluyveromyces lactis GN=ISU1 PE=3 SV=1 length=1041 |
|  |  |
| (O) 7:00 Vs 11:00 Vs 15:00 Vs 19:00 | |
| isotig | Annotation |
| isotig54164 | sp\|Q8W493\|FNRL2_ARATH Ferredoxin--NADP reductase, leaf isozyme 2, chloroplastic OS=Arabidopsis thaliana GN=LFNR2 PE=1 SV=1 length=755 |
|  |  |
| (P) 7:00 Vs 11:00 Vs 15:00 Vs 19:00 Vs 7:00-Next day | |
| isotig | Annotation |
| isotig56650 | NO_ANNOT_AVAILABLE length=612 |
| isotig69058 | NO_ANNOT_AVAILABLE length=287 |
| isotig00109 | sp\|Q6Z248\|HOX20_ORYSJ Homeobox-leucine zipper protein HOX20 OS=Oryza sativa subsp. japonica GN=HOX20 PE=2 SV=1 length=794 |
|  |  |
| (Q) 11:00 Vs 15:00 Vs 19:00 Vs 7:00-Next day | |
| isotig | Annotation |
| contig08982 | NO_ANNOT_AVAILABLE |
|  |  |
| (R) 15:00 Vs 19:00 Vs 7:00-Next day | |
| isotig | Annotation |
| isotig56670 | sp\|P55857\|SMT3_ORYSJ Ubiquitin-like protein SMT3 OS=Oryza sativa subsp. japonica GN=SMT3 PE=3 SV=1 length=610 |
