# Supplementary material for: Diurnal biomarkers reveal key photosynthetic genes associated with increased oil palm yield
Source: PLoS One. 2019 Mar 11;14(3):e0213591. doi: 10.1371/journal.pone.0213591 (PMC6411157; doi:10.1371/journal.pone.0213591)
Supplement: S7 Table — (DOCX) [file pone.0213591.s010.docx]

**Supplementary table 7**

Gene Ontology enrichment analysis of individual diurnal time point

07:00

| **Gene_ Ontology** | **Up_ regulate** | **Down_ regulate** | ***p*-value** |
| --- | --- | --- | --- |
| GO:0044237 | 6 | 0 | 0.01431 |
| GO:0009536 | 5 | 0 | 0.02535 |
| GO:0009507 | 4 | 0 | 0.0455 |
| GO:0044238 | 4 | 0 | 0.0455 |
| GO:0071704 | 4 | 0 | 0.0455 |

11:00

| **Gene_Ontology** | **Up_ regulate** | **Down_ regulate** | ***p-*value** |
| --- | --- | --- | --- |
| GO:0044444 | 9 | 1 | 0.01141 |
| GO:0005737 | 9 | 1 | 0.01141 |
| GO:0044424 | 9 | 1 | 0.01141 |
| GO:0005622 | 9 | 1 | 0.01141 |
| GO:0044464 | 9 | 1 | 0.01141 |
| GO:0005623 | 9 | 1 | 0.01141 |
| GO:0009536 | 6 | 0 | 0.01431 |
| GO:0009987 | 6 | 0 | 0.01431 |
| GO:0043231 | 8 | 1 | 0.01963 |
| GO:0043227 | 8 | 1 | 0.01963 |
| GO:0043229 | 8 | 1 | 0.01963 |
| GO:0043226 | 8 | 1 | 0.01963 |
| GO:0009507 | 5 | 0 | 0.02535 |
| GO:0044237 | 5 | 0 | 0.02535 |
| GO:0016020 | 4 | 0 | 0.04550 |
| GO:0051704 | 4 | 0 | 0.04550 |
| GO:0044249 | 4 | 0 | 0.04550 |
| GO:1901576 | 4 | 0 | 0.04550 |
| GO:0044699 | 4 | 0 | 0.04550 |
| GO:0044238 | 4 | 0 | 0.04550 |
| GO:0071704 | 4 | 0 | 0.04550 |

15:00

| **Gene_ Ontology** | **Up_ regulate** | **Down_ regulate** | ***p*-value** |
| --- | --- | --- | --- |
| GO:0003824 | 5 | 0 | 0.02535 |

19:00

| **Gene_ Ontology** | **Up_ regulate** | **Down_ regulate** | ***p*-value** |
| --- | --- | --- | --- |
| GO:0009536 | 10 | 1 | 0.00666 |
| GO:0032991 | 7 | 0 | 0.00815 |
| GO:0044237 | 15 | 4 | 0.01162 |

07:00

No significant enriched GO was observed.
